# Cooperative Binding of Substrate and Ions Drives Forward Cycling of the Human Creatine Transporter-1

**DOI:** 10.3389/fphys.2022.919439

**Published:** 2022-06-28

**Authors:** Clemens V. Farr, Ali El-Kasaby, Fatma A. Erdem, Sonja Sucic, Michael Freissmuth, Walter Sandtner

**Affiliations:** Institute of Pharmacology and the Gaston H. Glock Research Laboratories for Exploratory Drug Development, Center of Physiology and Pharmacology, Medical University of Vienna, Vienna, Austria

**Keywords:** creatine, creatine transporter-1, solute carrier 6/SLC6, concentrative power, cooperative binding, kinetic modeling, monocarboxylate transporter-12

## Abstract

Creatine serves as an ATP buffer and is thus an integral component of cellular energy metabolism. Most cells maintain their creatine levels via uptake by the creatine transporter (CRT-1, SLC6A8). The activity of CRT-1, therefore, is a major determinant of cytosolic creatine concentrations. We determined the kinetics of CRT-1 in real time by relying on electrophysiological recordings of transport-associated currents. Our analysis revealed that CRT-1 harvested the concentration gradient of NaCl and the membrane potential but not the potassium gradient to achieve a very high concentrative power. We investigated the mechanistic basis for the ability of CRT-1 to maintain the forward cycling mode in spite of high intracellular concentrations of creatine: this is achieved by cooperative binding of substrate and co-substrate ions, which, under physiological ion conditions, results in a very pronounced (i.e. about 500-fold) drop in the affinity of creatine to the inward-facing state of CRT-1. Kinetic estimates were integrated into a mathematical model of the transport cycle of CRT-1, which faithfully reproduced all experimental data. We interrogated the kinetic model to examine the most plausible mechanistic basis of cooperativity: based on this systematic exploration, we conclude that destabilization of binary rather than ternary complexes is necessary for CRT-1 to maintain the observed cytosolic creatine concentrations. Our model also provides a plausible explanation why neurons, heart and skeletal muscle cells must express a creatine releasing transporter to achieve rapid equilibration of the intracellular creatine pool.

## Introduction

The amino acid derivative creatine is present in abundant amounts in skeletal and cardiac muscle and in the nervous system of vertebrates. It is phosphorylated by creatine kinase to creatine phosphate, which allows for rapid regeneration of ATP at intracellular locations distant from mitochondria and thus for temporal and spatial ATP buffering (Wyss and Kaddurah-Daouk, 2000). Most cells do not synthesize creatine but rely on creatine uptake; thus, total creatine content is determined by the rate of creatine influx and the rate of creatine degradation. Influx of creatine from the blood or the cerebrospinal fluid is maintained by the creatine transporter (CRT-1, solute carrier-6 family member A8/SLC6A8) against a large concentration gradient. The importance of CRT-1 in preserving intracellular creatine content is evident from the consequences of its dysfunction in creatine transporter deficiency (Braissant and Henry, 2008; Farr et al., 2020): creatine transporter deficiency arises from point mutations in the coding sequence of CRT-1; the vast majority of these mutations lead to folding deficiency of the transporter (El-Kasaby et al., 2019; Bhat et al., 2021a). The affected patients suffer from intellectual disability and - depending on the nature of the mutation - from additional neurological symptoms including epileptic seizures, autism and hyperactivity (Farr et al., 2020). Breakdown of creatine occurs as cyclization to creatinine at a daily rate of about 2% in mammals (Borsook and Dubnoff, 1947; Picou et al., 1976). Because this reaction is driven by non-enzymatic conversion (Borsook and Dubnoff, 1947), it cannot be subject to biological regulation. As a consequence, CRT-1 is predicted to be the major determinant of cytosolic creatine concentrations in cells, which do not synthesize creatine. This prediction is supported by circumstantial evidence (Wyss and Kaddurah-Daouk, 2000).

CRT-1 is a member of the solute carrier 6 (SLC6) family of secondary active transporters. SLC6 transporters utilize the electrochemical gradient of co-substrate ions to fuel substrate uptake. In the case of human CRT-1, 2 Na^+^ and 1 Cl^-^ ions are thought to be translocated with each molecule of creatine (Dai et al., 1999). This stoichiometry defines the extent to which CRT-1 can harness the energy stored in the respective electrochemical gradients and determines the maximum achievable creatine concentration in the cytosol. However, as substrate accumulates within the cell, intracellular rebinding of substrate progressively slows the forward transport mode and thus leads to factual stagnancy of uptake long before the steady-state is reached. In many transporters, this rebinding is immaterial, because the influx of substrate is coupled to its enzymatic modification. In these instances, transporters and modifying enzymes are organized in complexes, termed transporter metabolons (Moraes and Reithmeier, 2012). Alternatively, solute carriers, which retrieve neurotransmitters at the plasma membrane, operate in relay with vesicular transporters. Accordingly, under physiological conditions, these plasmalemmal neurotransmitter transporters do not face high intracellular levels of substrate. They do not require a mechanism, which safeguards against their switching from the forward cycle into the exchange mode. In fact, this switch can be readily triggered in the monoamine transporters of the SLC6 family: when challenged with amphetamines, the transporters for dopamine, serotonin and noradrenaline mediate efflux of their cognate substrates because of the break imposed on the forward cycle mode (Hasenhuetl et al., 2018). Creatine is a remarkable osmolyte, because it can accumulate to very high levels within cells, e.g., about 7 mM in skeletal muscle (Kushmerick et al., 1992) and about 5 mM in neurons (Rackayova et al., 2017). Extracellular concentrations are in the range of 30–100 µM in peripheral blood and 17–90 µM in cerebrospinal fluid (Braissant and Henry, 2008). Thus, CRT-1 must operate in the forward cycle mode in spite of millimolar substrate concentrations on the intracellular side. The thermodynamic equilibrium predicts that CRT-1 can concentrate creatine up to >3000-fold and thus to intracellular levels substantially higher than those, which are observed at steady state. In the present study, we characterized the transport cycle of the creatine transporter to understand the binding mechanisms and their impact on transport kinetics. We relied on recording transport-associated currents with the patch clamp technique, which allows for probing the transport cycle with high temporal resolution and which affords control over intracellular composition and membrane voltage. The information extracted from these recordings were incorporated into a kinetic model, which emulated all relevant experimental findings. The calculations also provided two major insights: 1) a cooperative binding of substrate and co-substrate ions, which stabilizes ternary and quaternary complexes, is required to maintain the forward cycle mode in the presence of high intracellular creatine. 2) Cells, which accumulate intracellular creatine concentrations in the mM range, require an efflux transporter to support rapid equilibration.

## Materials and Methods

### Materials

Human embryonic kidney 293 (HEK293) cells were obtained from American Type Culture Collection (CRL-1573, Manassas, Virginia). Buffers and salts - with the exception of Mg(CH3COO^−^)_2_ and KH_2_PO_4_, which were from Merck KGaA (Darmstadt, Germany), cell culture media, penicillin-streptomycin stock solution (10.000 units/mL = 6 mg/ml penicillin and 10 mg streptomycin/mL), Hanks’s balanced salt solution, creatine monohydrate, β-guanidinopropionic acid (β-GPA) and cyclocreatine were purchased from Sigma-Aldrich (St. Louis, Missouri); 2-amino-1,4,5,6-tetrahydropyrimidine-5-carboxylic acid (ATPCA) was supplied by AKos Consulting and Solutions GmbH (Steinen, Germany). Fetal bovine serum (FBS) was from Capricorn Scientific (Ebsdorfergrund, Germany). The transfection reagents jetPRIME^®^ and polyethylenimine (linear 25 kDa) were purchased from Polyplus-transfection (New York City, New York) and from Santa Cruz Biotechnology (Dallas, Texas), respectively. The cDNA encoding the human creatine transporter-1/CRT-1 (transcript variant 1; SC116601) was originally bought from Origene (Rockville, Maryland) and inserted into the pEYFP-N1 vector from Clontech (Mountainview, California) to yield a version harboring YFP at the C-terminus (El-Kasaby et al., 2019) [^3^H]Creatine (creatine [N-methyl-^3^H], specific activity 75 Ci/mmol) and scintillation mixture (Rotiszint^®^ eco plus) were purchased from American Radiolabelled Chemicals (St. Louis, Missouri) and from Carl Roth GmbH (Karlsruhe, Germany), respectively. The rabbit polyclonal anti-GFP antibody (ab290) and horseradish peroxidase–linked anti-rabbit IgG1 antibody were purchased from Abcam (Cambridge, United Kingdom) and from Amersham Biosciences (Little Chalfont, United Kingdom). All chemicals were of analytical grade.

### Cell Culture and Transfections

HEK293 cells were cultured in Dulbecco’s modified Eagle’s medium containing high glucose (4.5 g/L = 25 mM), L-glutamine (584 mg/L = 4 mM), 10% FBS, 0.6 g/L penicillin and 0.1 g/L streptomycin in a humidified atmosphere containing 5% CO_2_ at 37°C. For electrophysiological recordings cells were split every 2–4 days to maintain subconfluent cultures (confluency <50%). The cells were transiently transfected with a plasmid encoding C-terminally YFP-tagged human CRT-1 with jetPRIME^®^ (3 µg DNA/6 cm dish) and polyethylenimine (20 µg DNA/15 cm dish) for patch clamp experiments and for uptake experiments according to the manufacturer’s protocol respectively. These ratios gave comparable transfection efficiency. One day after the transfection, cells were seeded at low density onto 3.5 cm dishes pre-coated with poly-D-lysine (PDL) for electrophysiological measurements. Similarly, 24 h after transfection, cells were seeded onto PDL-coated 48-well (10^5^ cells/well) or 96-well (36.000 cells/well) plates for uptake inhibition and steady state uptake experiments, respectively.

### Whole-Cell Patch Clamp Recordings

Sixteen to 24 h after seeding, currents were recorded from single cells in the whole-cell patch clamp configuration at 20–24 C. Cells were maintained in an aqueous external buffer (standard external solution) composed of 140 mM NaCl, 2.5 mM CaCl_2_, 2 mM MgCl_2_, 20 mM glucose and 10 mM HEPES (pH adjusted with NaOH to pH 7.4). In experiments designed to examine the dependence of current amplitude on co-substrate ions, Na^+^ and Cl^−^ in the external solution were individually replaced with equimolar N-methyl-D-glucamine.chloride (NMDGCl) and 2-(N-morpholino)ethanesulfonic acid. sodium (MESNa), respectively (the pH was adjusted with NMDG and NaOH, respectively). The standard internal solution in the micropipette contained 5.9 mM NaCl, 1 mM CaCl_2_, 0.7 mM MgCl_2_, 10 mM EGTA, 10 mM HEPES, and 133 mM K-gluconate (pH adjusted with KOH to pH 7.2). For a K^+^-free internal solution, K-gluconate was isoosmotically replaced by NMDG-MES (pH adjusted with NMDG). To study the intracellular effects of co-substrate ions, internal solutions with the following adjustments were prepared: 1) sole increase in Na^+^-concentration (133 mM MESNa, 6 mM NaCl, 1 mM CaCl_2_, 0.7 mM MgCl_2_, 10 mM EGTA, 10 mM HEPES, pH adjusted with NaOH to pH 7.2); 2) isolated increase of Cl^−^-concentration (139 mM NMDGCl, 1 mM CaCl_2_, 0.7 mM MgCl_2_, 10 mM EGTA, 10 mM HEPES, pH adjusted with NMDG to pH 7.2); 3) concomitant elevation of Na^+^ and Cl^−^ concentrations (139 mM NaCl, 1 mM CaCl_2_, 0.7 mM MgCl_2_, 10 mM EGTA, 10 mM HEPES, pH adjusted with NaOH to pH 7.2); 4) absence of Na^+^ and near-absence of Cl^−^ (139 mM NMDG-MES, 1 mM CaCl2, 0.7 mM Mg(CH3COO)_2_, 10 mM EGTA, 10 mM HEPES, pH adjusted with NMDG to pH 7.2). The composition of the solutions given in the figures reflect the ionic concentrations after pH adjustment.

In experiments, where the intracellular creatine concentration was raised, it was added to the standard internal solution and to the internal solution devoid of Na^+^ and Cl^−^ at the indicated concentrations. Osmolality was maintained constant by appropriate reduction in K-gluconate or in NMDG-MES.

Substrates of CRT-1 were applied using an Octaflow II system (ALA Scientific Instruments, Farmingdale, New York) and a Quartz Micromanifold (8 tubes with an inner diameter of 100 µM for application and a ninth tube with an inner diameter of 200 µm for flushing; fluorinated ethylene-propylene tubings) (ALA Scientific Instruments Inc., Farmingdale, New York). Currents were recorded via an Axopatch 200B amplifier (MDS Analytical Technologies, Sunnyvale, California) coupled with pClamp 10.2 software (Molecular Devices, LLC, San José, California) and digitized at 10 kHz using a Digidata 1440A data acquisition system (MDS Analytical Technologies). Recordings were filtered at 100 Hz and analyzed with Clampfit 10.2 software (Molecular Devices, LLC).

### Cellular Uptake of [^3^H]creatine

Twenty-four hours after seeding, cells were washed twice with Hank’s balanced salt solution and incubated therein for 30 min at 37°C and under 5% CO_2_. Subsequently, the solution was replaced by Krebs-HEPES buffer (10 mM HEPES. NaOH, 4.7 mM KCl, 2.2 mM CaCl_2_, 1.2 mM MgSO_4_, 10 mM glucose, 120 mM NaCl, pH = 7.4). In uptake inhibition experiments, logarithmically spaced concentrations of competing substrates were added, the reaction was initiated by adding [^3^H]creatine (1 μM, specific activity adjusted to 0.75 Ci/mmol with unlabeled creatine) and allowed to proceed for 6 min at 37°C. Steady state accumulation of substrate was measured with 10 nM and 100 µM [^3^H]creatine (with specific activities of 75 Ci/mmol and 7.5 mCi/mmol, respectively); the incubation lasted for up to 300 min at 37°C. Non-specific uptake was defined in the presence of 300 µM β-GPA. Assays were done in triplicate. The uptake reaction was terminated by aspiration of the solution and two rapid washes with ice cold Krebs-HEPES buffer. Cells were lysed in 1% SDS, the lysates were transferred to scintillation vials and the radioactivity content was determined by liquid scintillation counting.

### Membrane Preparation and Immunoblots

Membranes were prepared from transiently transfected HEK293 cells expressing YFP-tagged human CRT-1 or stably transfected HEK293 expressing YFP-tagged the human serotonin transporter (SERT) (Esendir et al., 2021) as follows: cells were washed twice with ice-cold PBS, mechanically detached and osmotically ruptured in a buffer consisting of 20 mM Tris. HCl (pH 7.5), 2 mM MgCl_2_ and protease inhibitors (Roche Complete™, Roche, Basel, Switzerland). The particulate material was harvested by centrifugation (15,000 g for 20 min at 4°C). The resulting pellet was resuspended in the same buffer and subjected to two freeze-thaw cycles (in liquid nitrogen followed by rapid thawing). Subsequently, samples were sonicated on ice twice for 7 s with a sonifier cell disruptor B15 (Branson Ultrasonics, Brookfield, Connecticut) at 50% intensity and centrifuged again at 15,000 g for 20 min at 4°C. The pellet was resuspended in the same buffer at a protein concentration of 1–5 mg/ml. Aliquots of this membrane samples (containing up to 10 µg of protein) were denatured in reducing Laemmli sample buffer (32.9 mM Tris. HCl (pH 6.8), 13% glycerol, 1% SDS, 0.01% bromophenol blue and 5% β-mercaptoethanol) for 30 min at 30°C, sonicated for 5 s in an ultrasonic water bath and incubated at 45°C for another 60 min. Proteins were separated by denaturing polyacrylamide gel electrophoresis (resolving gel 12% monomer concentration) and transferred onto polyvinylidene fluoride membranes. These were blocked with 5% non-fat dry milk (Blotto, ChemCruz™, Santa Cruz Biotechnology, Dallas, Texas) in Tris-buffered saline (pH = 7.5) containing 0.1% Tween 20 (TBST) and then incubated with a rabbit polyclonal antibody directed against GFP overnight at 4°C. After three additional washes, the immunoreactivity was visualized by chemoluminescence using a horseradish peroxidase-conjugated secondary antibody (1:5,000). An antiserum, which recognizes all G protein β-subunits (Hohenegger et al., 1996), was used for immunostaining to independently confirm the relative ratios of loaded samples.

### Kinetic Modeling

A kinetic model of CRT-1 was built based on the reaction scheme outlined in Figure 7A. Time-dependent changes in state occupancies were evaluated by numerical integration of the resulting system of differential equations using the Systems Biology Toolbox (Schmidt and Jirstrand, 2006) in Matlab 2019b (The MathWorks, Inc., Natick, Massachusetts). Voltage dependent transitions were modelled according to (Läuger, 1991) assuming a symmetric barrier as k_ij_ = k_ij_^0^exp (-z_Qi,j_FV/2RT), with k_ij_^0^ describing the rates at 0 mV, -z_Qi,j_ representing the net charge transferred during the transition, F the Faraday constant, V the membrane voltage, R the ideal gas constant and T the temperature. Coupled membrane currents were calculated as I = (-F∑z_Q,ij_ (p_i_k_ij_ - p_j_k_ji_))NC/N_A_, where NC is the number of transporters and N_A_ is the Avogadro constant. The extra- and intracellular ion concentrations were set to the values used in patch clamp experiments. The substrate concentration in the unit cell was calculated as described in (Burtscher et al., 2019). The creatinine sink in the unit cell (i.e. the conversion of creatine to creatinine) was modeled as d (creatine_in_ in Mol)/dt = [creatine_in_]*k_conversion_*N_A_*Volume_in_ where k_conversion_, N_A_ and Volume_in_ were 2.34*10^–7^ s^−1^, the Avogadro constant and 1 picoliter, respectively. Creatine efflux via MCT-12 was modeled as d (creatine_in_ in Mol)/dt = V_max_*([creatine_in_]/([creatine_in_ ]+K_M_))/N_A_, where K_M_ was 0.5 mM and N_A_ the Avogadro constant. V_max_ was adjusted such that the steady state concentration of intracellular creatine in the unit cell leveled out at 7 mM. This was achieved with V_max_ = 3.5*10^5^ molecules*s^−1^, if the unit cell expressed 5*10^5^ CRT-1 molecules. We note that V_max_ is the product of the number of MCT-12 units expressed in the unit cell and the turnover rate of this transporter.

### Statistics

Data from saturation and inhibition experiments were fitted by curvilinear regression to the equation for a rectangular hyperbola or to the Hill equation using GraphPad Prism 6 (Graphpad Software, San Diego, California) or Sigma Plot 12.0 (Systat Software Inc (San Jose, California). The more complex model (i.e. the Hill equation) was considered to be superior, if it significantly improved the fit, which was assessed by an F-test based on the extra sum of square principle. Current decays were fitted to the equation for a mono-exponential decay. Statistical tests were non-parametric and two-tailed, i.e. Mann-Whitney U-test and Kruskal–Wallis test for comparisons of two groups and ≥3 groups, respectively. Dunn’s multiple comparison was used as post hoc-test for comparing individual groups.

## Results

### Substrate Induced Currents Through CRT-1

**FIGURE 1:**
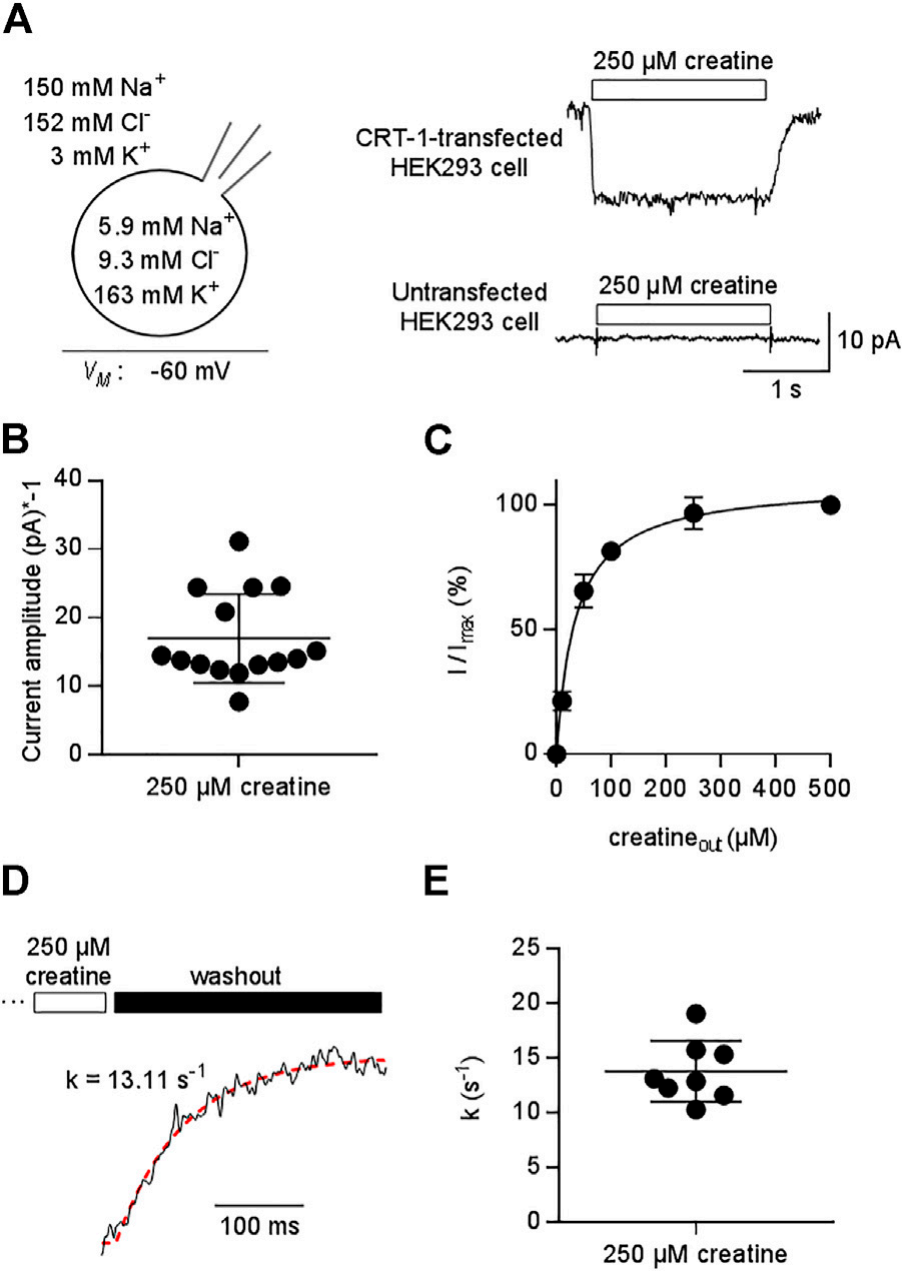
Patch clamp-recordings of creatine-induced currents through CRT-1 expressed in HEK293 cells. **(A)** The schematic representation on the left hand side illustrates the recording condition, i.e. the membrane potential, the extracellular and the intracellular ion concentrations employed in panels **(A–E)**. Right hand side: Representative current traces elicited by 250 µM creatine in a transiently transfected HEK293 cell expressing human CRT-1 (top) and in an untransfected cell (bottom). **(B)** Amplitude of CRT-1 mediated currents elicited by creatine: recordings were done as in panel A in 15 individual HEK293 cells transiently expressing human CRT-1. **(C)** Concentration-dependent increase in current amplitude through CRT-1 evoked by extracellular creatine (250 µM): recordings were done as in panel A with HEK293 cells transiently expressing CRT-1. For each cell, currents (I) were normalized to the current obtained with 500 µM creatine (I_max_) to correct for variable amplitude (see panel B). Data are means ± S.D. from 12 individual cells. The solid line was drawn by fitting the data to the equation for a rectangular hyperbola, which yielded an EC_50_ of 34.8 ± 1.9 µM. **(D)** Representative trace illustrating the deactivation of the substrate-induced current: the current through CRT-1 was elicited by 250 µM creatine as in panel A. Only the current decay is shown, which was evoked by solution exchange and by the thus resulting washout of creatine. The red dashed line was drawn by subjecting the data points to the equation for a mono-exponential decay. The rate constant k (13 s^−1^) extracted from the fitted curve provides an estimate for the turnover rate of CRT-1. **(E)** The rate constants of current decay were determined as illustrated in panel D from the recordings of 8 individual cells, the current amplitude of which are shown in panel B.

We relied on the whole-cell patch clamp configuration to record currents through CRT-1 transiently expressed in HEK293 cells. This approach allows for control of membrane voltage and of the ionic compositions on the extra- and intracellular side via the bath and the pipette solution, respectively. The upper right panel of Figure 1A shows a representative current trace elicited by rapid application of 250 µM creatine under physiological conditions (indicated in the left panel of Figure 1A). The substrate was applied for a period of 3 s with the membrane potential held at -60 mV. Creatine evoked an inwardly directed current, which persisted as long as it was present but vanished upon removal of substrate from the bath solution. This current was absent in untransfected HEK293 cells (lower right panel in Figure 1A). Creatine is a zwitterion. Hence, the inward current is due to the movement of net charges arising from the co-transported substrate ions.

As expected for a transient transfection, there was some variability in the current amplitude: when measured at -60 mV from different cells, the amplitude of the current induced by 250 µM creatine ranged from -7.77 pA to -31.13 pA (mean ± S.D. -16.9 ± 7.5 pA , Figure 1B). We accounted for this variability by normalizing the current amplitude to the maximum current elicited by 500 µM creatine. It is evident from Figure 1C that this concentration was close to saturation: an EC_50_ of 34.8 ± 1.9 was estimated for creatine by fitting the concentration-dependent increase in the normalized current to a rectangular hyperbola.

Removal of substrate allows for exit of the transporter from the transport cycle. The rate of current deactivation provides an estimate for the turnover rate of the transporter (Erdem et al., 2019). We extracted this rate by a fitting a mono-exponential function to the current decay (Figure 1D): on average, the rate was approximately 15 s^−1^ (Figures 1D,E). Thus, at a membrane potential of -60 mV, one transporter can carry about 15 creatine molecules through the membrane within one second.

### Substrate-Induced Currents Through CRT-1 Are Strictly Coupled to Substrate Transport

**FIGURE 2:**
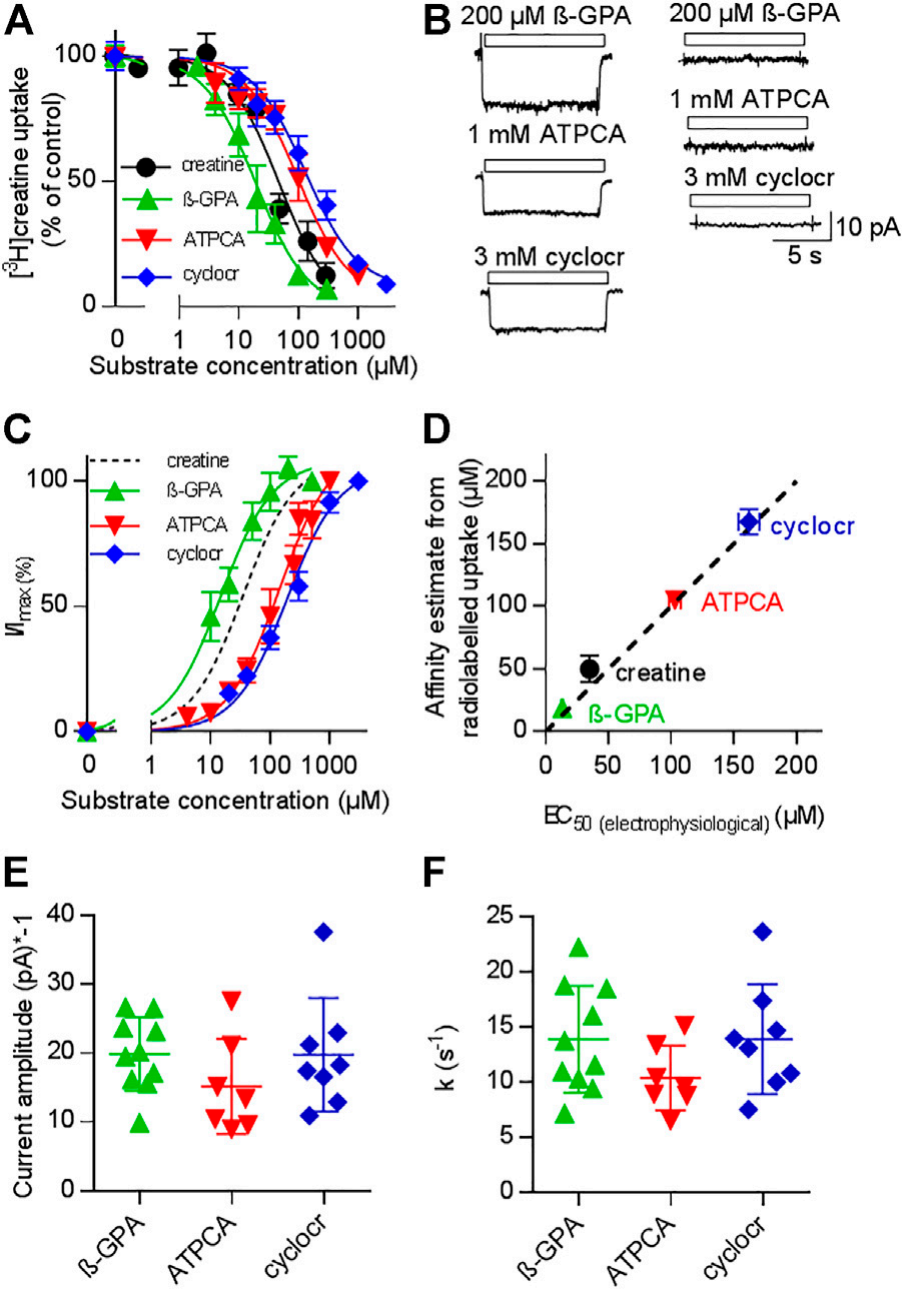
Comparison of uptake inhibition **(A,D)** and CRT-1 mediated currents **(B–F)** evoked by different substrates. **(A)** Uptake of [^3^H]creatine by HEK293 cells transiently expressing CRT-1 in absence and presence of increasing concentrations of CRT-1 substrates creatine (black circles), β-guaninidinopropionate (β-GPA, green upward triangles), 2-amino-1,4,5,6-tetrahydropyrimidine-5-carboxylic acid (ATPCA, red downward triangles) and cyclocreatine (cyclocr, blue diamonds). Specific [^3^H]creatine uptake in the presence of competing substrates was normalized to the uptake in their absence. This control uptake was 14.7 ± 1.7 pmol min^−1^.10^–6^ cells and set to 100% to account for inter-assay variations. The lines were drawn by fitting the data points to the equation for a monophasic inhibition curve. The concentrations required for half-maximal inhibition (IC_50_) were estimated as 18.8 ± 1.1 µM, 48.4 ± 7.0 µM, 105.0 ± 6.7 µM and 167.3 ± 9.9 µM for β-GPA, creatine, ATPCA and cyclocreatine respectively. K_i_-values are virtually equal to IC_50_-values, because the concentration of [^3^H]creatine (1 µM) was far below its K_M,_ Data are means from three independent experiments carried out in triplicate; error bars indicate S.D. **(B)** Representative current traces recorded from HEK293 cells transiently expressing CRT-1 (left hand graph) and from untransfected control HEK293 cells (right hand graph) when superfused with 200 µM β-GPA, 1 mM ATPCA and 3 mM cyclocreatine (cyclocr), respectively. **(C)** Concentration-dependent increase in current amplitude through CRT-1 evoked by superfusion with β-GPA, ATPCA and cyclocreatine (cyclocr): recordings were done as in panel B with HEK293 cells transiently expressing CRT-1. For individual cells, currents (I) were normalized to the current amplitude (I_max_) obtained with the near saturating concentrations of each compound employed in panel B. Data are means ± S.D. from 9, 9–16 and 7 individual cells for β-GPA, ATPCA and cyclocreatine, respectively. The solid lines were drawn by fitting the data to the equation for a rectangular hyperbola resulting in EC_50_ estimates of 13.19 ± 0.73 µM, 103 ± 4. µM; and 162.0 ± 8.49 µM for β-GPA, ATPCA and cyclocreatine, respectively. The dashed black line shows the position of the fitted curve for creatine taken from Figure 1C. **(D)** Correlation between EC_50_ for evoking currents and IC_50_ for inhibition of [^3^H]creatine uptake (r^2^ = 0.99, slope = 0.96 ± 0.05). The black dashed line represents the line of identity. **(E,F)** HEK293 cells transiently expressing CRT-1 were superfused with near-saturating concentrations of substrates, i.e. by 200 µM β-GPA (n = 10), 1 mM ATPCA (n = 7) and 3 mM cyclocreatine (n = 8) as in panel B to record the steady state currents and the time course of current decay upon washout of the substrate. The amplitude of the steady state currents and the rate constants of current decay are shown in panels E and F, respectively. In both instances, inter- and intragroup variation were comparable (Kruskal–Wallis test). The recording conditions (i.e. the membrane potential, the extracellular and the intracellular ion concentrations) employed in panels **(B–F)** were as indicated in Figure 1A.

If currents through a transporter are strictly coupled to substrate translocation, the K_M_ for substrate uptake and the EC_50_ for current induction by the substrate must be equivalent readouts of the transport cycle (Burtscher et al., 2019; Schicker et al., 2021). We verified that CRT-1 substrate-induced currents report faithfully on the transport cycle by comparing the potency of three different synthetic substrates in inhibiting uptake of [^3^H]creatine (Figure 2A) and in promoting a current through CRT-1 (Figures 2B,C): The concentration-dependent inhibition of [^3^H]creatine uptake by β-guanidinopropionic acid (β-GPA, green upward triangles in Figure 2A), creatine (black circles in Figure 2A), 2-amino-1,4,5,6-tetrahydropyrimidine-5-carboxylic acid (ATPCA, red downward triangles in Figure 2A) and cyclocreatine (blue diamonds in Figure 2A) was adequately described by a monophasic inhibition curve; the affinity estimates (IC_50_) extracted from the fit were 18.8 ± 1.1 µM, 48.4 ± 7.0 µM, 105.0 ± 6.7 µM and 167.3 ± 9.9 µM for β-GPA, creatine, ATPCA and cyclocreatine, respectively.

Because the concentration of the radiotracer (1 µM) was far below its K_M_, the IC_50_ is virtually identical to the K_i_. The left panel of Figure 2B shows representative current traces, which were elicited in HEK293 cells expressing CRT-1: it is evident that, at concentrations which were close to saturation (i.e. corresponding to ≥10*K_i_), β-GPA (upper trace), ATPCA (middle trace) and cyclocreatine (bottom trace) elicited currents through CRT-1, which were of similar shape and magnitude. These currents were absent in untransfected control cells (right panel in Figure 2B). In Figure 2C we plotted the normalized current amplitudes as a function of the concentration of β-GPA (green upward triangles), ATPCA (red downward triangles) and cyclocreatine (blue diamonds): the rank order of potency for induction of current through CRT-1 matched that for uptake inhibition (cf. Figures 2A,C). In fact, in a plot of the IC_50_-estimates for uptake inhibition over the EC_50_-values for current induction, the data points for the different substrates fell onto a line with a slope (slope 0.96 ± 0.05) not significantly different from 1 and thus reasonably close to the line of identity (Figure 2D). We also verified that the current amplitudes were of similar magnitude: Figure 2E shows the current amplitudes recorded from different cells after application of β-GPA, ATPCA and cyclocreatine at concentrations close to saturation. Finally, we analyzed the time course of current deactivation on removal of β-GPA, ATPCA and cyclocreatine: the extracted rates did not differ in a statistically significant manner (Figure 2F) and they were comparable to the rates determined for the cognate substrate creatine (cf. Figure 1E). Taken together these data show that 1) β-GPA, ATPCA and cyclocreatine are full substrates, 2) the current-induced by substrate is strictly coupled to substrate translocation, 3) neither binding of substrate nor its intracellular release is the rate-limiting step in the transport cycle, because the deactivation proceeds at equivalent rates in spite of vastly different affinities.

### The Stoichiometry of CRT-1

**FIGURE 3:**
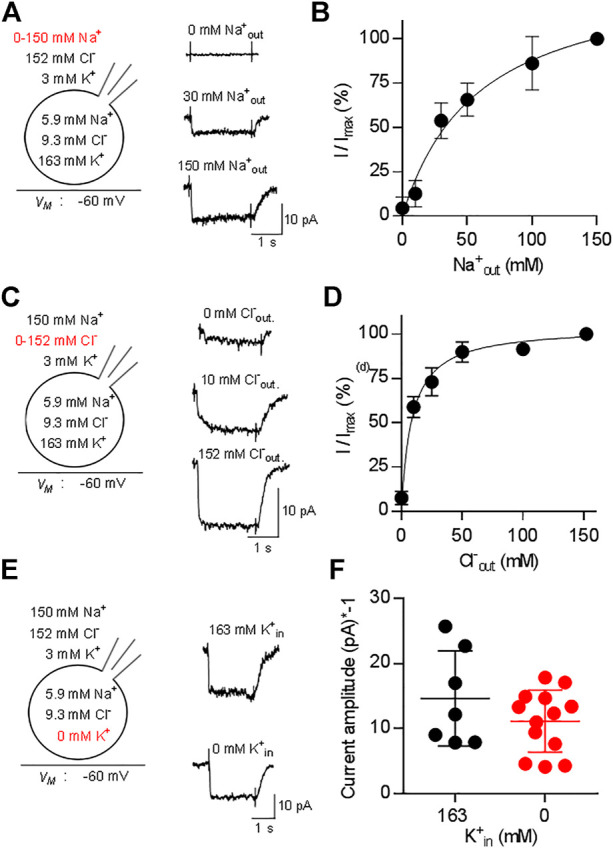
Dependence of currents through CRT-1 on physiologic ion gradients. The schematic representation on the left hand side of each row illustrates the recording condition, i.e. the membrane potential, the extracellular and the intracellular ion concentrations employed in panels **(A–F)**; the experimental variation in ionic composition is indicated in red. Panels A, C, E show the original recordings of currents through CRT-1, which were elicited by superfusion of HK293 cells transiently expressing CRT-1 with 250 µM creatine in the presence of 0 mM, 30 and 150 mM extracellular Na^+^ **(A)**, or in the presence of 0 mM, 10 and 152 mM extracellular Cl^−^ **(C)** or in the presence 163 mM and absence of intracellular K^+^ **(E)**. Panels B and D show the dependence of creatine-induced current amplitudes on the concentration of extracellular Na^+^ panel **(B)** and Cl^−^ **(D)** (n = 15) during superfusion with 250 µM creatine: currents were recorded at the indicated ion concentrations as in panels A and C; the current amplitude (I) was normalized to the amplitude of the steady state current measured at the highest concentration of Na^+^ or Cl^−^ (I_max_) to account for variation in transporter expression and cell size. The solid line were drawn by fitting the data to the Hill equation **(B)** or to a rectangular hyperbola **(D)**. In panel B, the data points were more adequately described by the Hill equation than by the equation for a rectangular hyperbola (F-test, *p* = 0.012), providing an EC_50_ estimate for sodium of 37.1 ± 4.2 µM and a Hill coefficient of 1.41 ± 0.17 for sodium. In panel D, the Hill equation did not provide a better fit than the rectangular hyperbola (F-test, *p* = 0.082). The EC_50_ estimate for Cl^−^ was 8.6 ± 0.9 µM. Data are means ± S.D., n = 12 and 14 in panels B and D respectively. Panel F compares the amplitudes of currents, which were elicited by 250 µM creatine in the presence of 163 mM cytosolic K^+^ (n = 7) or in absence of intracellular K^+^ (n = 13) and recorded as in panel **(E)**. Omitting intracellular potassium from the internal solution did not have any significant effect on current amplitude (*p* = 0.483, Mann-Whitney test).

We addressed the ionic requirements for the transport cycle by recording the substrate-induced current through CRT-1 by varying the concentrations of external Na^+^ (Figure 3A,B) and Cl^−^ (Figures 3C,D). The representative traces, which were sequentially recorded from the cell, illustrate that, in the absence of external Na^+^, creatine (250 µM) failed to elicit any current (upper trace in Figure 3A) and that the current amplitude increased with rising external Na^+^ (middle and bottom trace in Figure 3A). A plot of the normalized current amplitude over the Na^+^ concentration yielded a curve, which was adequately described by the Hill-equation (Figure 3B): an EC_50_ value and a Hill-coefficient of 37.1 ± 4.2 mM and 1.4 ± 0.2, respectively, were estimated from the fit. The calculated Hill-coefficient is consistent with the co-transport of two Na^+^ ions with one substrate molecule in each transport cycle. We note that the Hill coefficient, though significantly larger than 1, is less than 2. Modeling shows that a Hill coefficient of 2 can only be achieved, if one assumes that the two Na^+^ ions bind with exactly the same affinities. If Na1 binds faster than the Na2 (or vice versa), the Hill coefficient drops below 2. In contrast, in the nominal absence of external chloride, application of 250 µM creatine elicited consistently a small, but detectable current (upper trace in Figure 3C). The current amplitude increased with rising external Cl^−^ (Figure 3C, middle and bottom trace from the same cell). A plot of the normalized current amplitudes as a function of the external Cl^−^ concentration was described by a rectangular hyperbola (Figure 3D) resulting in an EC_50_ estimate of 8.6 ± 0.9 mM for extracellular Cl^−^. The deactivation rate also contains transitions of the transporter from earlier states in the transport cycle. These are negligible, if the transporter operates at maximum speed, but they become limiting e.g., at low co-substrate concentrations. Thus, the model predicts slowed deactivation at low Na^+^ or low Cl^−^. This was, in fact, observed: The deactivation rate k in the presence of 30 mM Na^+^ and of 10 mM Cl-was 5.4 ± 1.2 s^−1^ and 6.2 ± 1.8 s^−1^, respectively, and thus about half of that seen at saturating co-substrate ions (k = 12.5 ± 4.9 s^−1^). This difference was statistically significant (*p* < 0.05 Kruskal–Wallis test followed by Dunn’s multiple comparisons) and the decline in deactivation was commensurate with the decline in current amplitude.

Some solute carriers utilize the energy contained in the transmembrane gradient of potassium to increase their concentrative power. This is also true for the serotonin transporter (SLC6A4), a relative of CRT-1 (Hasenhuetl et al., 2016; Bhat et al., 2021b). Accordingly, we examined the effect of intracellular K^+^ on substrate-induced currents carried by CRT-1 by recording currents elicited by 250 µM creatine in the presence and absence of 163 mM internal K^+^. Representative traces are shown in Figure 3E to illustrate that removal of K_in_^+^ did not affect the kinetics of the current; recordings from different cells verified that the average current amplitude was comparable in the absence and presence of internal K^+^ (Figure 3F). Hence, we conclude that the intracellular K^+^ concentration and thus the K^+^ gradient is immaterial to CRT-1. Taken together our findings are, therefore, consistent with the previously proposed transport stoichiometry for CRT-1 (Dai et al., 1999), i.e., in each cycle CRT-1 translocates two sodium ions and one chloride ion through the membrane with one molecule of creatine. We note, though, that electrophysiological measurements alone cannot provide the unequivocal identification of the transported ion species.

### The Substrate-Induced Current Through CRT-1 Is Voltage-dependent

**FIGURE 4:**
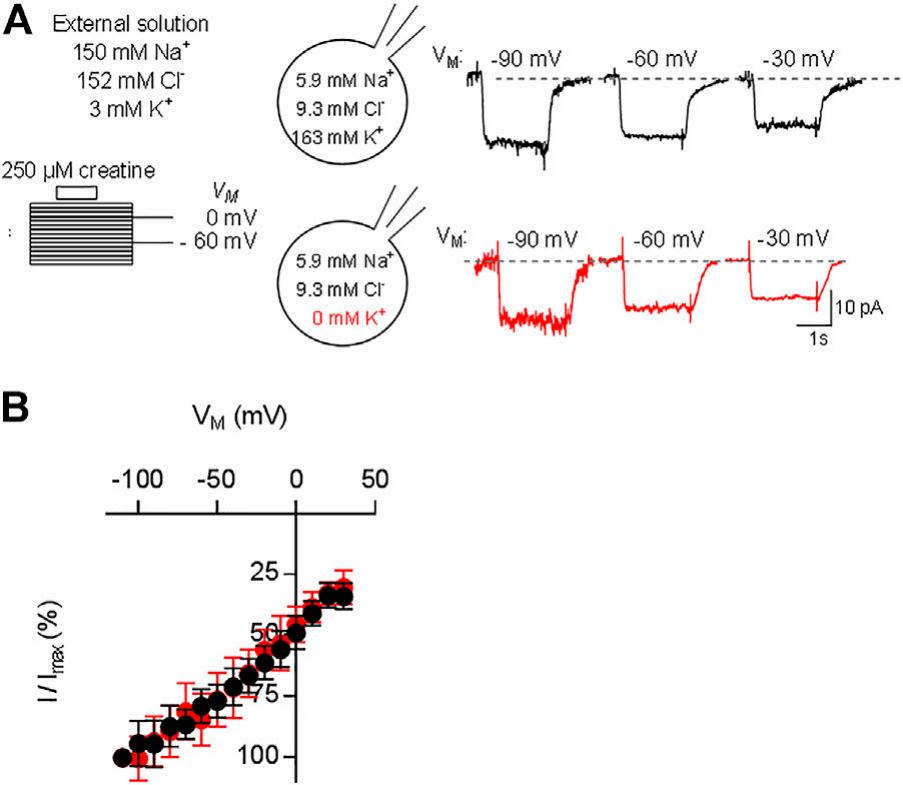
Current-voltage relation of CRT-1. A&B: HEK293 cells transiently expressing CRT-1 were clamped at membrane potentials ranging from −110 mV to +30 mV for 15 s. During each step 250 µM creatine was applied for 2 s. In panel **(A)**, the schematic representation on the left hand side illustrates the recording condition, i.e. the extracellular and the intracellular ion concentrations and the voltage steps. Representative traces were recorded at −90, −60 and −30 mV in cells filled with a pipet solution containing 163 mM K^+^ (top row, right hand side of panel A) or NMDG (bottom row, right hand side of panel A). Data from 12 independent recordings (means ± S.D.) in the presence of 163 mM intracellular K^+^ (black circles) and the absence of intracellular K^+^ (red circles) are summarized in panel **(B)** for the indicated voltage range. Current amplitudes at each voltage (I) were normalized to the current amplitude recorded at -110 mV (I_max_) to account for cell-to-cell variation in expression levels.

The transport stoichiometry of CRT-1 predicts that one positive net-charge is moved through the membrane in each transport cycle. Accordingly, the substrate uptake rate by CRT-1 is predicted to be voltage-dependent. In addition, the data summarized in Figure 3F predict that the K^+^ gradient does not affect the current-voltage relation. Both predictions were verified by examining the voltage-dependence of the substrate uptake rate: we measured the current amplitudes induced by 250 µM creatine at potentials ranging from -110 to 30 mV (the protocol is illustrated in the scheme of Figure 4A): representative current traces recorded at −90 mV, −60 mV and −30 mV are shown in Figure 4A in the presence (top row) and absence of intracellular K+ (bottom row) to illustrate the increase and decline in current amplitude at more negative and more positive membrane voltage, respectively. The normalized current amplitudes were plotted as a function of membrane voltage (Figure 4B). It is evident that over the range of voltage, which was investigated, the current-voltage relation is essentially linear and that potassium does neither affect its slope nor its position.

### Raising the Intracellular Concentrations of Substrate and Co-substrate Ions Reduces the Amplitude of Substrate-Induced Currents Through CRT-1

**FIGURE 5:**
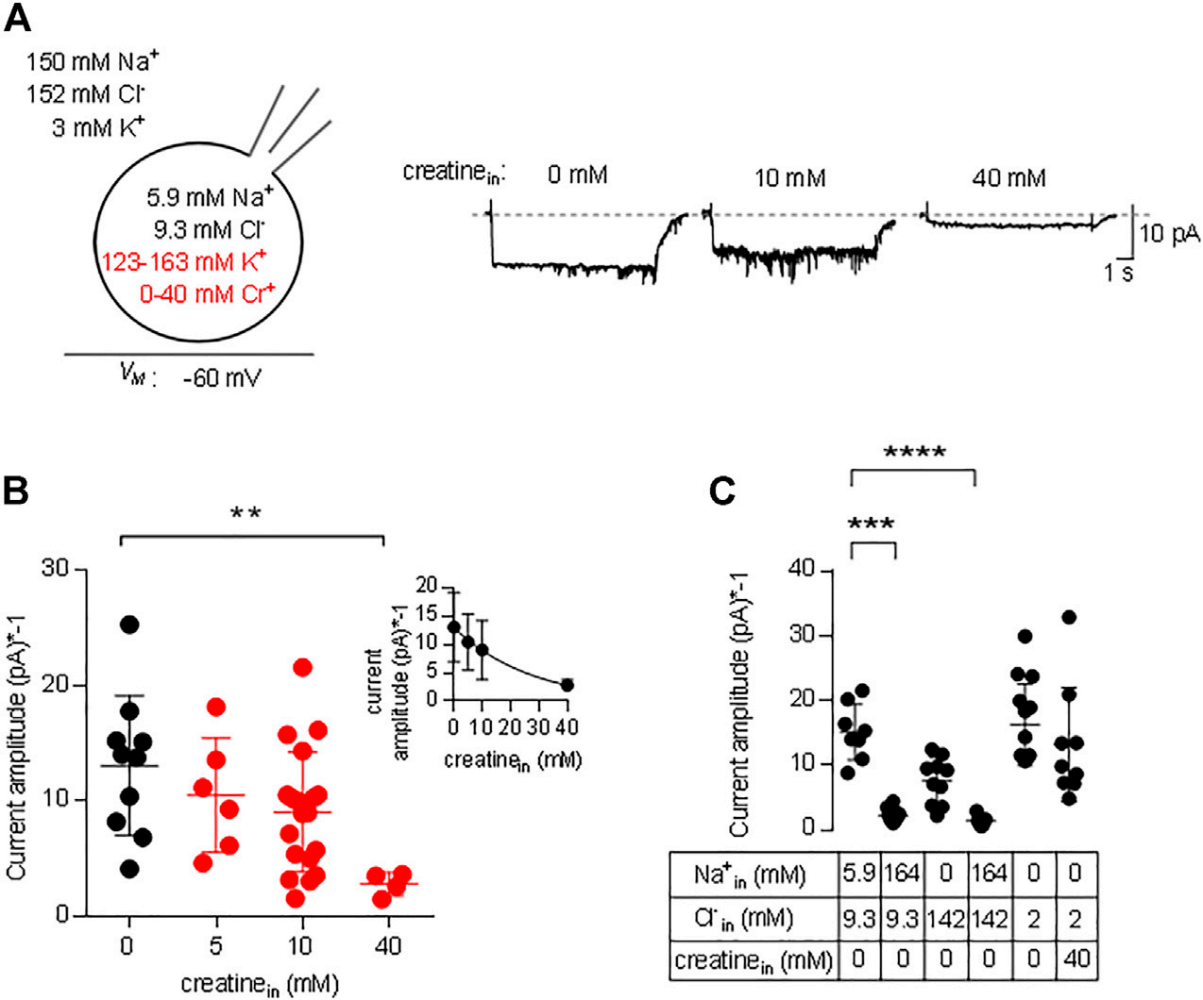
Inhibition by intracellular creatine of currents through CRT-1. **(A)** The schematic representation on the left hand side illustrates the recording condition, i.e. the extracellular and the intracellular ion concentrations, the variation in intracellular creatine concentration (highlighted in red) and the membrane potential employed in panels A and B. The representative traces shown on the right hand side were recorded from HEK293 cells transiently expressing CRT-1. Currents were elicited by superfusion with 250 µM creatine from cells, which had been filled with an internal solution containing 0 mM, 10 and 40 mM creatine. **(B)** Amplitudes of the steady state current, which was recorded as in panel A in the absence (n = 10) and presence of 5 mM (n = 6), 10 mM (n = 6), 20 mM (n = 19) and 40 mM (n = 5) intracellular creatine. Shown are individual values and means ± S.D. The difference between 0 and 40 mM intracellular creatine was statistically significant (Kruskal–Wallis test followed by Dunn’s post hoc test; *p* = 0.0055). Inset: The data were also fitted to the equation for a monophasic inhibition; the resulting estimate for the IC_50_ was 16.2 ± 4.3 mM intracellular creatine. **(C)** Currents were recorded in HEK293 cells transiently expressing CRT-1, which had been filled with the indicated concentrations of Na^+^, Cl^−^ and/or creatine via the recording pipet. Steady state currents were elicited by superfusion with 250 µM creatine at −60 mV; their amplitudes are shown as individual values (n = 8, 10, 10, 7, 14 and 9 from left to right) and as means ± S.D. Sole elevation of intracellular Na^+^ (to 140 mM) resulted in a significant decrease in current amplitude (*p* ≤ 0.001 vs control internal solution with 5.9 mM Na^+^ and 9.3 mM Cl^−^, Kruskal–Wallis test followed by Dunn’s post hoc test); this statistically significant reduction in current amplitude was also seen, if both Na^+^ and Cl^−^ were raised to 140 mM (*p* ≤ 0.0001).

In the transport cycle, the inward facing conformation releases the substrate (and co-substrates). However, substrate is prone to rebind as its intracellular concentration rises. Rebinding of substrate hampers progression of the transport cycle. Accordingly, there must be an inverse relation between substrate uptake rate and intracellular substrate concentration. We explored the effect of intracellular substrate on the transport cycle of CRT-1 by analyzing the steady current amplitude evoked by the application of 250 µM creatine in the absence and presence of intracellular creatine. Figure 5A shows representative traces, in which the internal creatine concentration was raised from 0 to 10 and 40 mM. The amplitude of the current was only modestly reduced at 10 mM creatine, but a pronounced decline was observed at 40 mM. Because the current amplitude depends on cell size and the expression levels of CRT-1, we averaged the current amplitudes from individual recordings of control cells (0 mM intracellular creatine) and cells, which had been filled with 5 mM, 10 and 40 mM creatine (Figure 5B) and plotted these average amplitudes as a function of internal substrate concentration (inset to Figure 5B). The resulting curve was adequately described by the equation for a monophasic inhibition. From the fitted curve, we estimated that 16.2 ± 4.3 mM internal creatine was required for half-maximum inhibition of the substrate-induced net inward current.

We also examined the effect of intracellular Na^+^ on the amplitude of currents through CRT-1 induced by 250 µM creatine. Similar to intracellular creatine, Na_in_^+^ is also expected to rebind to the inward facing transporter at high concentrations and to thereby hamper progression through the transport cycle (Hasenhuetl et al., 2016; Hasenhuetl et al., 2018; Erdem et al., 2019). In Figure 5C, we compared the current amplitudes in the presence of 5.9 and 164 mM Na_in_^+^. At 164 mM Na_in_^+^ the current amplitude was significantly reduced, both in the presence of 142 and 9.3 mM Cl_in_^−^ (second and fourth data set in Figure 5C). We also tested separately the effect of Cl_in_^−^ on the amplitudes of the currents elicited by the application of 250 µM creatine: in the presence of 142 mM Cl_in_^−^ (and in the nominal absence of intracellular Na^+^), there wasn’t any statistically significant suppression (third data set in Figure 5C) of the amplitude compared to the standard internal solution (first data set in Figure 5C) or to 2 mM Cl^−^ in the recording pipette (fifth data set in Figure 5C). Most importantly, in the nominal absence of intracellular sodium and in the presence of 2 mM intracellular chloride, 40 mM intracellular creatine failed to affect the amplitude of the net inward current (*cf*. fifth and sixth data set in Figure 5C). Thus, the inhibitory action of intracellular creatine is contingent on the intracellular presence of co-substrate ions, which support rebinding of creatine the inward-facing state of CRT-1 and thus preclude progression through the transport cycle.

### Cooperative (co)-Substrate Binding to CRT-1

**FIGURE 6:**
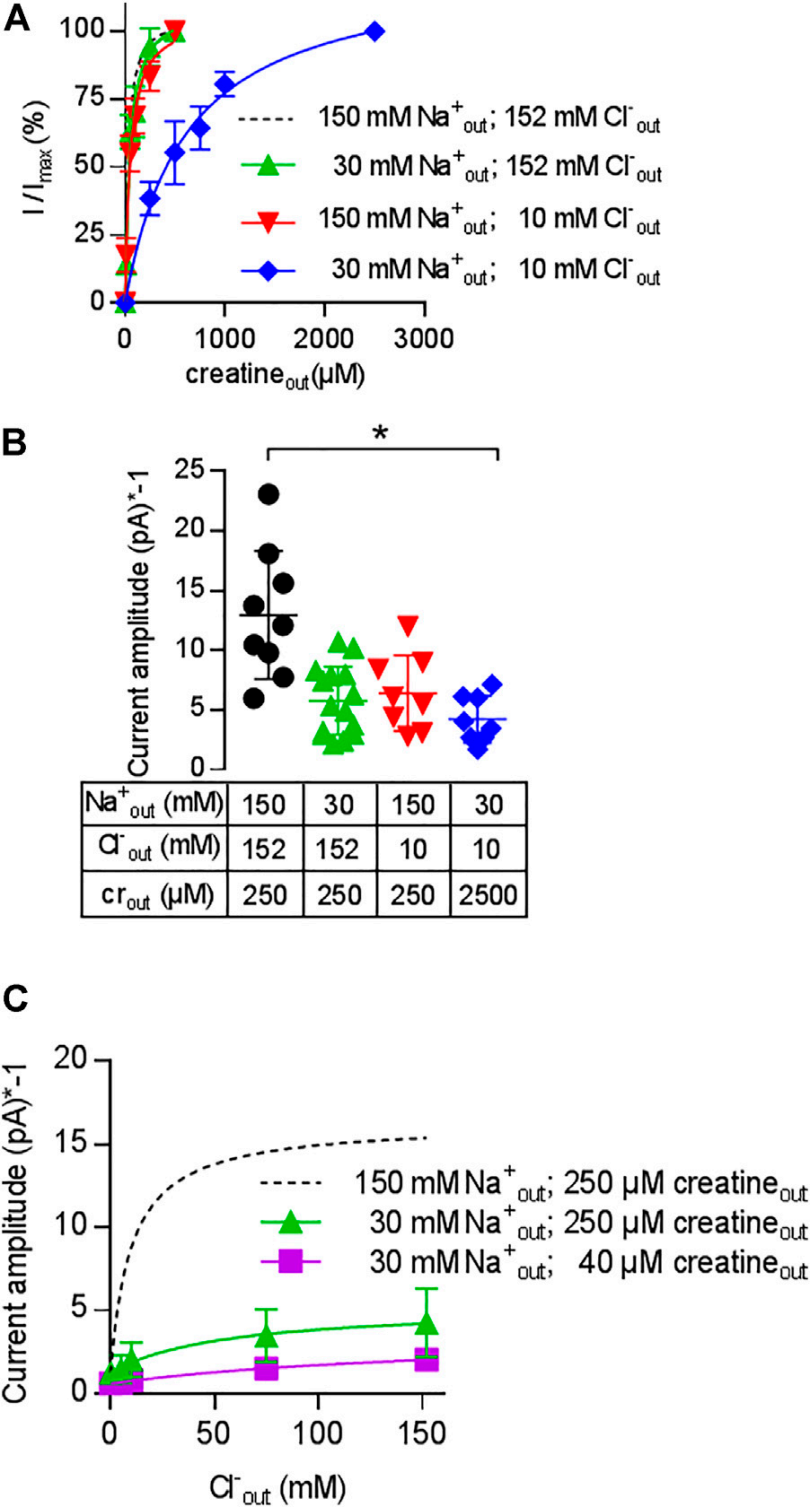
Cooperativity of creatine, sodium and chloride. A&B: Change in the EC_50_ of creatine **(A)** and in current amplitude **(B)** resulting from reduction in the concentration of co-substrate ions: transport-associated currents were elicited by superfusing HEK293 cells transiently expressing CRT-1 with an external solution containing increasing concentrations of creatine and 30 mM Na^+^ and 152 mM Cl^−^ (green upward triangles), 152 mM Na^+^ and 10 mM Cl^−^ (red downward triangles) or 30 mM Na^+^ and 10 mM Cl^−^ (blue diamonds). In panel A, the current amplitude (I) at each creatine concentration was normalized to the amplitude observed at the highest creatine concentration (I_max_, recorded at 250 µM or 2.5 mM creatine for the triangles and diamonds, respectively). The solid lines were drawn by fitting the data to the equation for a rectangular hyperbola. EC_50_-values were estimated as 47.2 ± 5.0 µM (low Na^+^ = 30 mM Na^+^ and 152 mM Cl^−^), 40.1 ± 6.4 µM (low Cl^−^ = 152 mM Na^+^ and 10 mM Cl^−^) and 609.9 ± 63.5 µM (low NaCl = 30 mM Na^+^ and 10 mM Cl^−^). Data are means ± S.D. from 6, 8 and 8 independent recordings for low Na^+^ (= 30 mM Na^+^ and 152 mM Cl^−^), low Cl^−^ (=152 mM Na^+^ and 10 mM Cl^−^) and low NaCl (= 30 mM Na^+^ and 10 mM Cl^−^) respectively. For the sake of comparison, the dashed line shows the concentration-response curve from Figure 1C, which was obtained in the presence of 152 mM external NaCl. Panel B shows the current amplitudes obtained at the maximum concentrations of creatine (i.e. 250 µM or 2.5 mM as indicated) and at the ionic conditions employed in panel A; current amplitudes were also recorded under standard external ionic conditions (i.e. 152 mM NaCl, black circles) for comparison; each symbol represents an individual cell (n = 9, 15, 8 and 8 from left to right). Means ± S.D. are also indicated. The current amplitude recorded in the presence of 30 mM Na^+^ and 10 mM Cl^−^ was significantly smaller than the control current amplitude recorded in the presence of 152 mM NaCl (*p < 0.05; Kruskal–Wallis test followed by Dunn’s post hoc test). **(C)** Shift in the EC_50_ of chloride upon reduction of external Na^+^ and creatine. Transport-associated currents were elicited by superfusing HEK293 cells transiently expressing CRT-1 with an external solution containing 40 µM (squares) or 250 µM creatine (circles and triangles), 30 mM (squares and triangles) or 150 mM Na^+^ (circles) and increasing concentrations of Cl^−^. The amplitudes of the steady currents are shown. The recordings in the presence of 250 µM creatine and 150 mM Na^+^ (dashed lines) are the same as in Figure 3D (with absolute rather than normalized values) and are shown here as reference. The solid lines were drawn by fitting the data to the equation for a rectangular hyperbola, which yielded EC_50_ estimates of 49.1 ± 10.9 mM and 172 ± 49.6 mM for 30 mM Na^+^/250 µM creatine, and 30 mM Na^+^/40 µM creatine, respectively; data are means ± S.D. from 6–13 independent recordings.

The results summarized in Figure 5 show that CRT-1 can undergo essentially unimpeded forward cycling and support substrate influx against an intracellular creatine concentration of up to >>1 mM. Half-maximum inhibition is seen at 16 mM intracellular creatine, i.e. at a concentration, which is about 500-fold higher than the K_M_ for creatine uptake from the extracellular side. This can only be achieved, if creatine is prevented from rebinding to the inward-facing conformation of CRT-1. Accordingly, the affinity of creatine for the inward facing conformation of CRT-1 must be low (i.e., much lower than its affinity for the outward facing conformation, for which the EC_50_ for current induction by the substrate and the K_M_ for uptake provide good estimates). Differences between intracellular and extracellular affinities can be achieved by cooperative binding of substrate and co-substrate to solute carriers: the substrate affinity is high when the co-substrates (i.e. Na^+^ and Cl^−^) are bound to the transporter but low in their absence (Hasenhuetl et al., 2018; Erdem et al., 2019; Hasenhuetl et al., 2019 ). Under physiological conditions, the extracellular concentrations of Na^+^ and Cl^−^ are much higher than those in the cytosol. As a consequence, the apparent substrate affinities to the outward and inward facing conformation of the transporter are high and low, respectively. We first examined, whether Na^+^ and creatine bound in a cooperative manner by comparing the EC_50_ for current induction by creatine in the presence of extracellular 30 mM Na^+^ (i.e. the EC_50_ of Na^+^ for supporting the substrate-induced current; cf. Figure 3B) and 150 mM extracellular Na^+^. It is evident from Figure 6A that in the presence of 30 mM extracellular Na^+^, the EC_50_ of creatine (47.2 ± 5.0 µM) for current induction was only reduced by a very modest extent (green upward triangles in Figure 6A) when compared to that seen in the presence of 150 mM Na^+^ (dashed black curve in Figure 6A). This unexpected observation was inconsistent with cooperative binding of Na^+^ and creatine. It raised the question, how a drop in intracellular substrate affinity can be achieved to levels low enough to prevent intracellular creatine from rebinding to CRT-1. In the experiments summarized in Figure 4B, both intracellular Na^+^ and Cl^−^ were present at low concentrations (i.e.; 5.9 mM Na^+^ and 9.3 mM Cl^−^). It was, therefore, conceivable that it was not substrate and Na^+^ but substrate and Cl^−,^ which bound in a cooperative manner. We explored this conjecture by determining the EC_50_ for current induction by creatine at 10 mM extracellular Cl^−^ (i.e. the EC_50_ of Cl^−^ for supporting the substrate-induced current; cf. Figure 3D). Again the shift in the EC_50_ of creatine (40.1 ± 6.4 µM) was minor (red downward triangles in Figure 6A) and hence did not substantiate a strongly cooperative binding of substrate and Cl^−^. Finally, we determined the EC_50_ for current induction by creatine by simultaneously lowering extracellular Cl^−^ and Na^+^, i.e. to 10 and 30 mM, respectively (blue diamonds in Figure 6A): this resulted in a substantial shift of the concentration-response curve of creatine (EC_50_ = 609.9 ± 63.5 µM). The curves in Figure 6A show normalized data, because this allows for readily appreciating the differences in apparent affinity. The individual and combined reduction in external co-substrate ion concentrations also lowers the occupancy of the transport competent state. Accordingly, we observed a decline in current amplitude, which was commensurate with the reduced occupancy (Figure 6B).

We further explored the cooperative nature of substrate and co-substrate binding by examining the effect of lowered levels of extracellular sodium and creatine on the concentration-response curve for chloride: in the presence of 30 mM Na^+^, there was shift in the EC_50_ of Cl^−^ (49.3 ± 1.0 mM) for supporting the creatine-induced current (triangles, Figure 6C). If the concentration of both, Na+ (30 mM) and creatine (40 µM) were reduced (squares, Figure 6C), there was a further decline in current amplitude and the EC_50_ was further shifted to the right. It is obvious that the EC_50_ estimate (172.0 ± 49.6 mM) was determined with poor precision, because the highest concentration of chloride was only in the range of the EC_50_. However, the magnitude of the shift was commensurate with the shift seen in Figure 6A. Taken together, these observations confirm that CRT-1 relies on a cooperative mechanism to adjust substrate affinity: the cooperativity requires the formation of a quaternary complex of CRT-1, substrate, sodium and chloride.

### Kinetic Model of CRT-1

**FIGURE 7:**
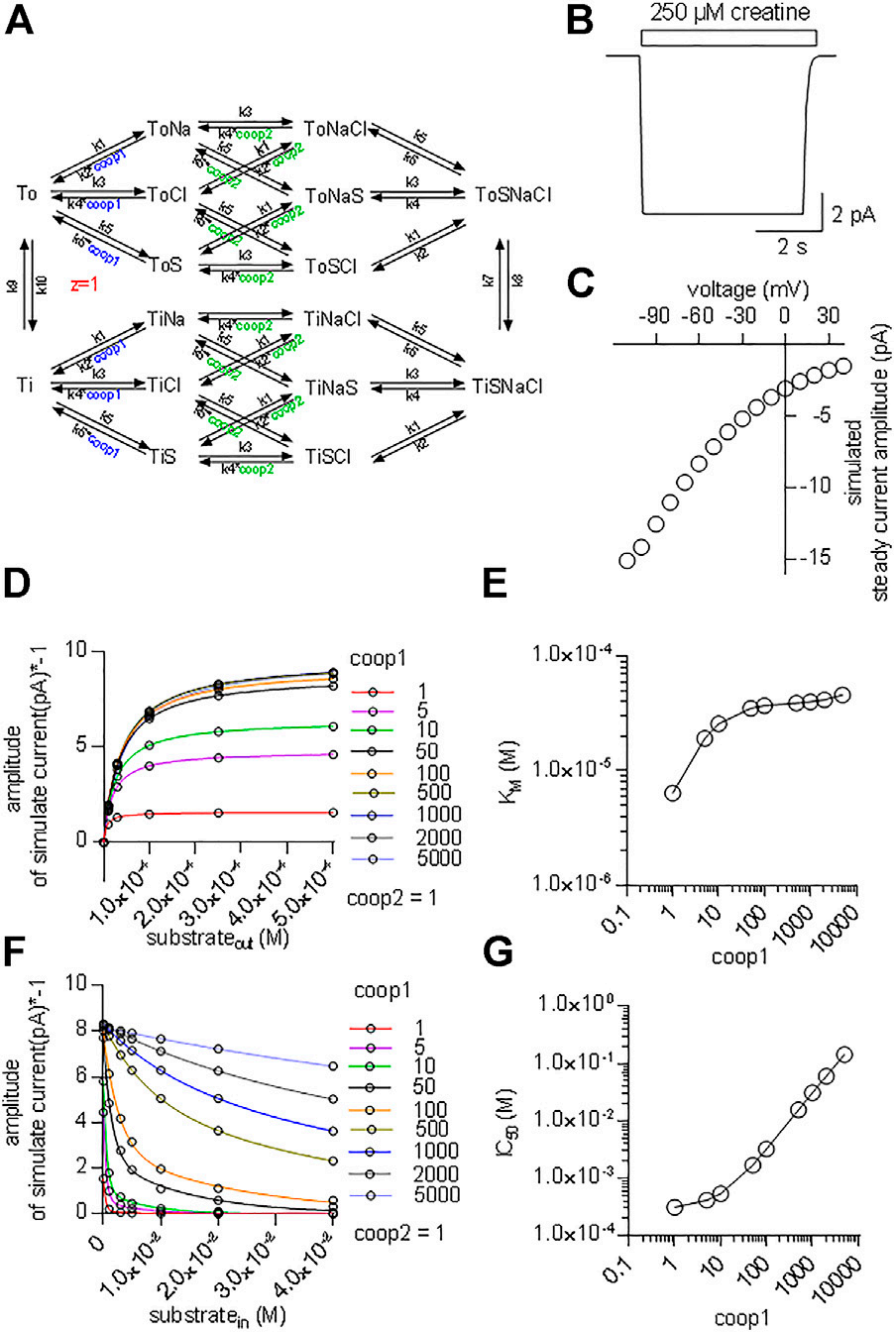
Kinetic model of CRT-1 with cooperativity favouring stable ternary complexes. **(A)** Reaction scheme of the transport cycle of CRT-1. The rate constants used to parameterize the model were as follows: k1 = 10^12^ M^−2^*s^−2^; k2 = 2.5*10^7^ s^−2^; k3 = 10^6^ M^−1^*s^−1^; k4 = 5*10^3^ s^−1^; k5 = 10^7^ M^−1^*s^−1^; k6 = 2,500 s^−1^; k7 = 100 s^−1^; k8 = 100 s^−1^; k9 = 5.2 s^−1^; k10 = 5.2 s^−1^. **(B)** The synthetic current trace elicited by the application of 250 µM creatine was generated by the model outlined in panel A with coop1 = 500 & coop2 = 1 using the experimental conditions (ion gradients and membrane potential) employed in Figure 1A. **(C)** Current-voltage relation of the creatine-induced steady current predicted by the model shown in panel A with coop1 = 500 & coop2 = 1. **(D)** The reaction scheme illustrated in Panel A was used to generate the synthetic concentration-response curves for currents elicited by external creatine. In the simulations, coop1 was varied from 1 to 5,000 (as indicated in the caption) with coop2 = 1. The solid lines were drawn by fitting the data points to the equation for a rectangular hyperbola. **(E)** The K_M_ values for substrate were extracted from the curve fitting shown in panel D and plotted as a function of the corresponding coop1 value. **(F)** Inhibition of the steady current amplitude as a function of the intracellular concentration of creatine. The different symbols show the results from simulations in which coop1 was varied from 1 to 5,000 (as indicated in the caption) with coop2 = 1. **(G)** The IC_50_ values for internal substrate were extracted from the curve fitting shown in panel C and plotted as a function of the corresponding coop1 value.

It is necessary to assume a random binding order for co-substrates and substrate to model cooperative binding (see reaction scheme in Figure 7A). The resulting scheme for CRT-1 includes three binary complexes (ToCl, ToNa and ToS), three ternary complexes (ToClNa, ToClS and ToNaS), the transport competent quaternary complex (ToClNaS) and all inward facing counterparts (i.e.,TiCl, TiNa etc.). For the sake of parsimony, we assumed that the two Na^+^ ions bind to CRT-1 in a single reaction. We accounted for cooperativity in (co)-substrate binding by introducing cooperativity factors, which allow for modeling accelerated release of a given (co)-substrate by multiplying the dissociation rate constant with the cooperativity factor: hence, assigning a value >1 to the cooperativity factor reduces (co)-substrate affinity. In the reaction scheme we introduced two cooperativity factors, coop1 and coop2. Changes in the value of coop1 and coop2 affect the stability of binary and ternary complexes, respectively.

Figure 7B shows a simulated current trace elicited on application of 250 µM creatine to a hypothetical cell held at −60 mV. The cell was assumed to express 5*10^6^ molecules of CRT-1 on its surface. This is a plausible number for a solute carrier, which is transiently expressed in a HEK293 cell (Li et al., 2015). The rate constants in the model were chosen such that the substrate turnover rate was 15/cycle at −60 mV. With this turnover rate and this number of CRT-1 molecules per cell the amplitude of the simulated current was approximately 10 pA. This is in reasonable agreement with the current amplitudes, which were observed in the actual experiments (cf. Figures 1A,B). In the model, we assumed that the rate limiting reaction in the transport cycle of CRT-1 is the return step of the substrate-free inward to the substrate-free outward facing conformation. We further assumed that one charge is moved through the membrane in this reaction. Accordingly, in our model, it is this charge translocation, which generates the current. We assigned voltage dependence to the return step and not to any other transition in the transport cycle, because this accounted for the observed current voltage relation (Figure 7C) and for the failure of CRT-1 to produce a transient current on rapid application of creatine.

Our experimental results indicate that both co-substrates must dissociate to reduce the affinity of creatine and to allow for continuous forward cycling of CRT-1 in the presence of high intracellular creatine (*cf.* Figures 5B,C). This implies that in CRT-1, the ternary complexes (e.g. ToClS, ToNaS) are stable but the binary complexes (TiS,TiNa) are not. This boundary condition was incorporated into the model by assigning coop2 a value of 1 and a larger numerical to coop1. With coop2 = 1, we ensured that the ternary complexes remained stable. A coop1 >1 forced the binary complexes to readily dissociate. We first explored systematically the effect of varying coop1 over a large range of numerical values (i.e. 1 to 5,000, Figure 7D). Current amplitude (Figure 7D) and K_M_ (Figure 7E) increased as coop1 was raised from 1 to 10. For the sake of simplicity, we refer to the EC_50_-values extracted from the fit of these curves as K_M_. This is justified, because these two parameters are equivalent (see above, Figure 2D). Both, current amplitudes (Figure 7D) and K_M_ values (Figure 7E) reached a plateau at about 8 pA and 40 μM, respectively with coop1>10. Thus, these experiments defined the lower boundary of cooperativity (reflecting rapid disassembly of binary complexes), which was required to allow for rapid cycling of CRT-1 in the forward transport mode and to emulate the experimentally measured K_M_ for creatine. However, it is not possible by this approach to further narrow down the range of cooperativity. This was achieved by simulating inhibition of substrate uptake by intracellular substrate with coop2 = 1 and variable coop1 (Figure 7F). It is evident from Figure 7F that the inhibition by intracellular substrate was relieved with increasing coop1. The concentrations giving half-maximum inhibition of transport-associated currents was plotted as a function of coop1: the resulting graph shows that rising coop1 afforded an infinite gain in eliminating substrate inhibition on the intracellular side (Figure 7G). It is also evident from Figure 7F that a coop1 of 500 recapitulates the experimental data shown in Figure 5B. In fact, the calculated IC_50_ of 15.7 mM closely matches the IC_50_ of 16.2 mM estimated from the experimental recordings.

**FIGURE 8:**
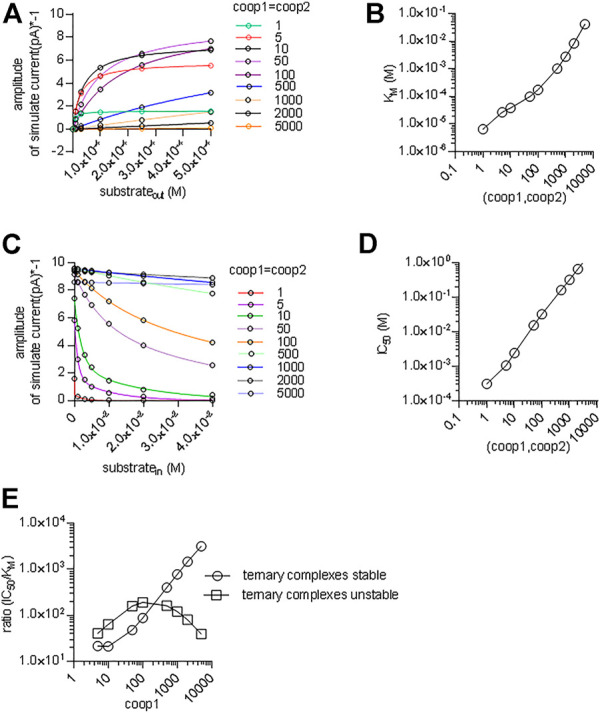
A cooperative model allowing for unstable ternary complex fails to support large enough a difference in the apparent substrate affinity for the outward and inward facing conformation. **(A)** The reaction scheme illustrated in Figure 7A was used to generate the synthetic concentration-response curves for currents elicited by external creatine. In the simulations, coop1 and coop2 were assumed to be equal and allowed to vary from 1 to 5,000 (as indicated in the caption). The solid lines were drawn by fitting the data points to the equation for a rectangular hyperbola. **(B)** The K_M_ values for substrate were extracted from the curve fitting shown in panel A and plotted as a function of the corresponding value of coop1 & coop2. **(C)** Inhibition of the steady current amplitude as a function of the intracellular concentration of creatine. The differently colored curves show the results from simulations in which coop1 and coop2 were assumed to be equal and allowed to vary from 1 to 5,000 (as indicated in the caption). **(D)** The IC_50_ values for internal substrate were extracted from the curve fitting shown in panel C and plotted as a function of the corresponding value of coop1 & coop2. **(E)** Plotted in the graph are the ratios (IC_50_/K_M_) from the data in panels B and D (open squares) and from Figures 7E,G (open circles) as a function of the coop 1 values used in the corresponding simulations, where coop 2 = coop1 and hence ternary complexes are unstable (open squares) or coop 2 = 1 and hence ternary complexes are stable (open circles).

We also interrogated the kinetic model to examine the alternative scenario, in which both binary and ternary complexes were assumed to be equally unstable by assigning them similar coop1 and coop2. In this instance, the synthetic current amplitudes were commensurate with the experimental data within the range of 5–100 for coop1 and coop2 (Figure 8A). However, the predicted K_M_ values for substrate uptake increased progressively with rising coop1 and coop2 (Figure 8B). Accordingly, the K_M_ was only in the range of about 30 µM with coop1 and coop2 equaling 5 and 10. However, if CRT-1 were to operate with these cooperativity factors of 5 and 10, intracellular creatine would inhibit the forward transport mode with IC_50_ values of 1 and 2 mM, respectively (Figures 8C,D). The experimentally determined IC_50_ of 16 mM (cf. Figure 5C) was recapitulated with coop1 and coop 2 equaling 50. We stress that, in the synthetic experiments of Figure 8C, the external substrate concentration had to be raised to 0.4 M (rather than the 250 µM of Figure 7D) to ensure saturation in the tested range of coop values. This adjustment was necessary to account for the high K_M_ values at large coop factors (*cf.* Figure 8A).

We calculated the ratios IC_50_/K_M_ for the two scenarios and plotted them as a function of the cooperativity factors (Figure 8E). The scenario, which posits variable coop 2 (and thus both, unstable binary and ternary complexes), results in a bell-shaped curve (squares in Figure 8E) with a maximum IC_50_/K_M_ ratio of 200. This ratio is substantially lower than the IC_50_/K_M_ ratio of 500, which was observed in the experimental recordings (cf. Figure 1C & Figure 5B). Importantly, the IC_50_/K_M_ ratio of 200 is too small to allow the K_M_ for substrate uptake to adopt values in the range of 30 µM and, at the same time, to allow for essentially unimpeded forward transport in the presence of millimolar intracellular substrate. Thus, this analysis supports the conclusion that the large IC_50_/K_M_ ratio observed in CRT-1 is contingent on the preferential stability of its ternary and rapid disassembly of its binary complexes.

**FIGURE 9:**
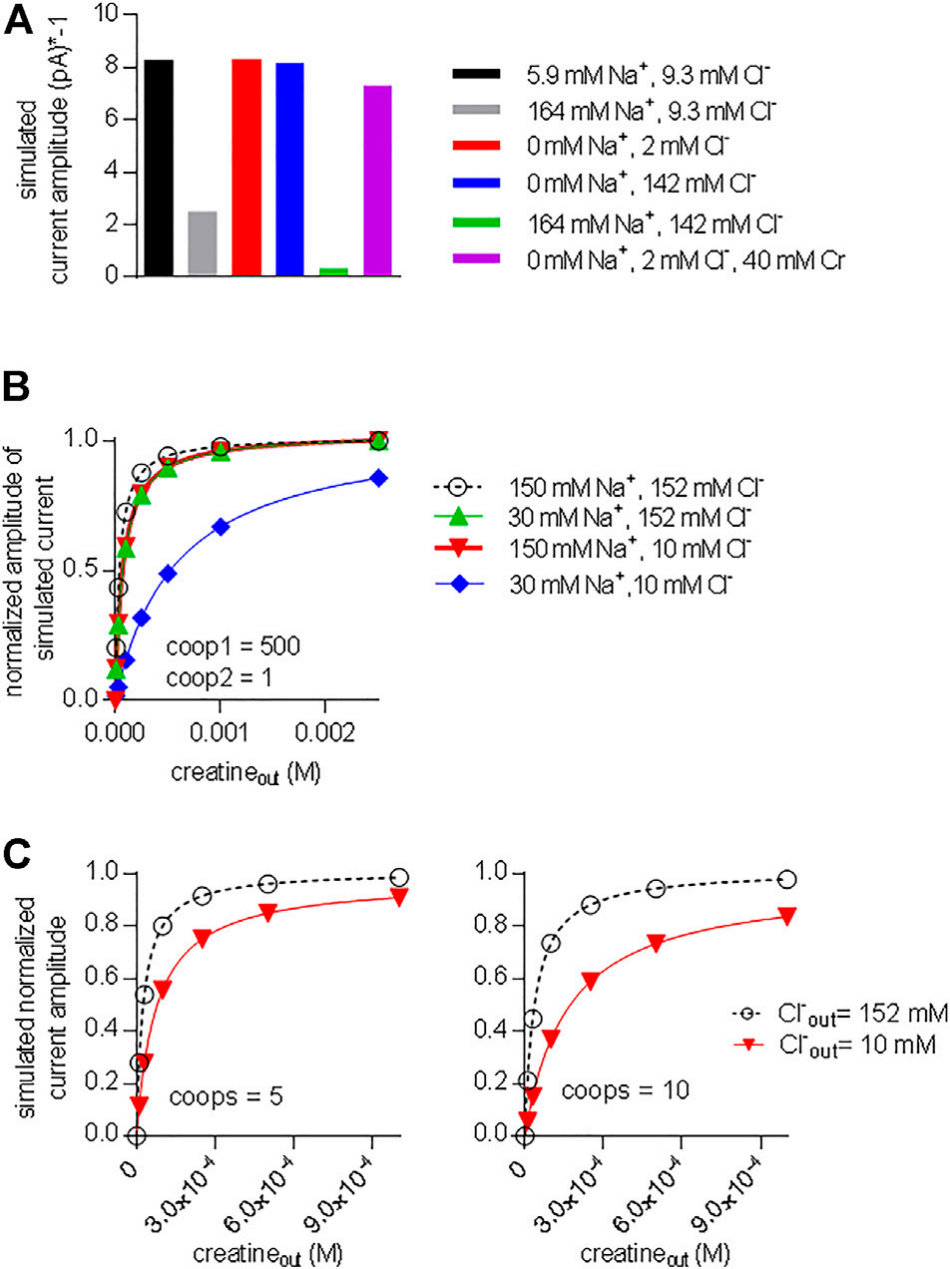
A model with stable ternary complexes recapitulates the cooperative action of co-substrate ions and on substrate-elicited currents. **(A)** Synthetic currents elicited by 250 µM extracellular creatine were generated using the kinetic model outlined in Figure 7A with coop1 = 500 and coop 2 = 1 in the presence of the indicated intracellular ionic concentrations. The amplitude of this synthetic steady current was plotted to highlight the inhibition by sole elevation of intracellular Na^+^ and the combined elevation of intracellular Na^+^ and Cl^−^. **(B)** Synthetic concentration-response curves for currents (shown as normalized amplitude of the steady current) elicited by external creatine in the presence of the indicated ions, which recapitulate the experimental conditions employed Figure 6A. **(C)** Synthetic data were generated in the presence of 150 mM Na^+^ and 152 mM Cl^−^ (black curve) and 150 mM Na^+^ and 10 mM Cl^−^ (red curve) by the kinetic model shown in Figure 7A with coop1 and coop2 = 5 (left hand graph) and coop 1 and coop2 = 10 (right hand graph). In these instances, lowering the extracellular concentration of only one co-substrate (i.e. Cl^−^: from 152 to 10 mM) was sufficient to produce a pronounced right shift of the EC_50_ value for current induction.

The acid test for the model illustrated in Figure 7A was to verify, if it was capable of reproducing additional key experimental observations with coop1 = 500 and coop2 = 1. 1) Synthetic data were generated with variations in the intracellular co-substrate concentrations: the substrate-induced current through CRT-1 was suppressed by solely increasing Na^+^ (grey bar in Figure 9A) but not Cl^−^ (blue bar in Figure 9A) on the intracellular side. Similarly, raising intracellular creatine in the absence of Na^+^ and Cl^−^ (red bar, Figure 9A) failed to suppress forward cycling of CRT-1. Thus, the synthetic data recapitulated the experimental data summarized in Figure 5C. 2) We also interrogated the model to address the cooperative binding of substrate and co-substrate ions to the outward facing state of CRT-1. In the presence of extracellular 150 mM Na^+^ and 152 mM Cl^−^, the model generates a concentration response curve for current induction by extracellular creatine (black circles in Figure 9B) with an EC_50_ of 40 µM extracted from the fitted curve (dashed line in Figure 9B). This EC_50_ is in reasonable agreement with the actual determined from the actual recordings (*cf*. Figure 1C). Based on the experimental finding (cf. Figure 3B,C), our kinetic model operates with EC_50_-values of 30 and 10 mM for extracellular Na^+^ and Cl^−^, respectively. Accordingly, we examined how lowering extracellular Na^+^ to 30 mM (in the presence of 152 mM external Cl^−^) affected the concentration-response curve for creatine (green upward triangles in Figure 9B): there was only a modest shift to the right (EC_50_ of creatine = 75.8 µM) extracted from the fitted curve (green line in Figure 9B). The magnitude of the shift was consistent with the experimental observation in Figure 6A. Similarly, we interrogated the model for the shift in EC_50_ of creatine resulting from lowering extracellular Cl^−^ to 10 mM (in the presence of 150 mM external Na^+^): this again resulted in a modest shift (EC_50_ of creatine 73.6 µM; downward triangles and red curve in Figure 9B). However, if the extracellular concentration of both co-substrates was lowered (i.e. to 30 mM Na^+^, 10 mM Cl^−^), the model produced a concentration-response curve for creatine, which was substantially shifted to the right (EC_50_ = 576.1 µM; blue diamonds and blue line in Figure 9B). Thus, a kinetic model, which relied on cooperativity factors (of coop1 = 500 and coop2 = 1) to account for continuous forward cycling of CRT-1 in the presence of high intracellular creatine concentrations, also faithfully recapitulated the affinity shifts, which were observed by varying the co-substrate concentrations on the on the extracellular side (cf. Figure 6A).

We also simulated the alternative scenario, which was examined in Figure 8 and in which we allowed for rapid disassembly of the ternary complexes by increasing the value of coop2. As mentioned above, a kinetic model, with coop 1 and coop2 in the range of 5–10 can emulate concentration-response curves for creatine-induced currents, which are reasonably similar to the experimental observations (see Figures 8A,B). In the left and right hand panel of Figure 9C, we assumed values of 5 and 10, respectively, for both coop1 and coop2. Thus we posited that binary and ternary complexes disassembled at equal rates. In the two panels, we compared the concentration-response curve for creatine (by plotting normalized current amplitudes) at 152 and 10 mM external Cl^−^ (and constant external Na^+^ = 150 mM). The simulations showed that a value of 5 for both coop factors sufficed to produce a substantial right shift in the EC_50_ of creatine (left hand panel in Figure 9C) and the shift was more pronounced with coop1 and coop2 equaling 10 (left hand panel in Figure 9C). However, in the actual experiments, sole lowering Cl^−^ to 10 mM did not result in any substantial shift in the saturation curve of creatine (cf. red downward triangles in Figure 6A). Thus, only the model, which assigned stability to the ternary complexes (with a coop1 = 500) rather than to the binary complexes, emulated all key experimental observations.

### Predicting the Intracellular Creatine Concentration

**FIGURE 10:**
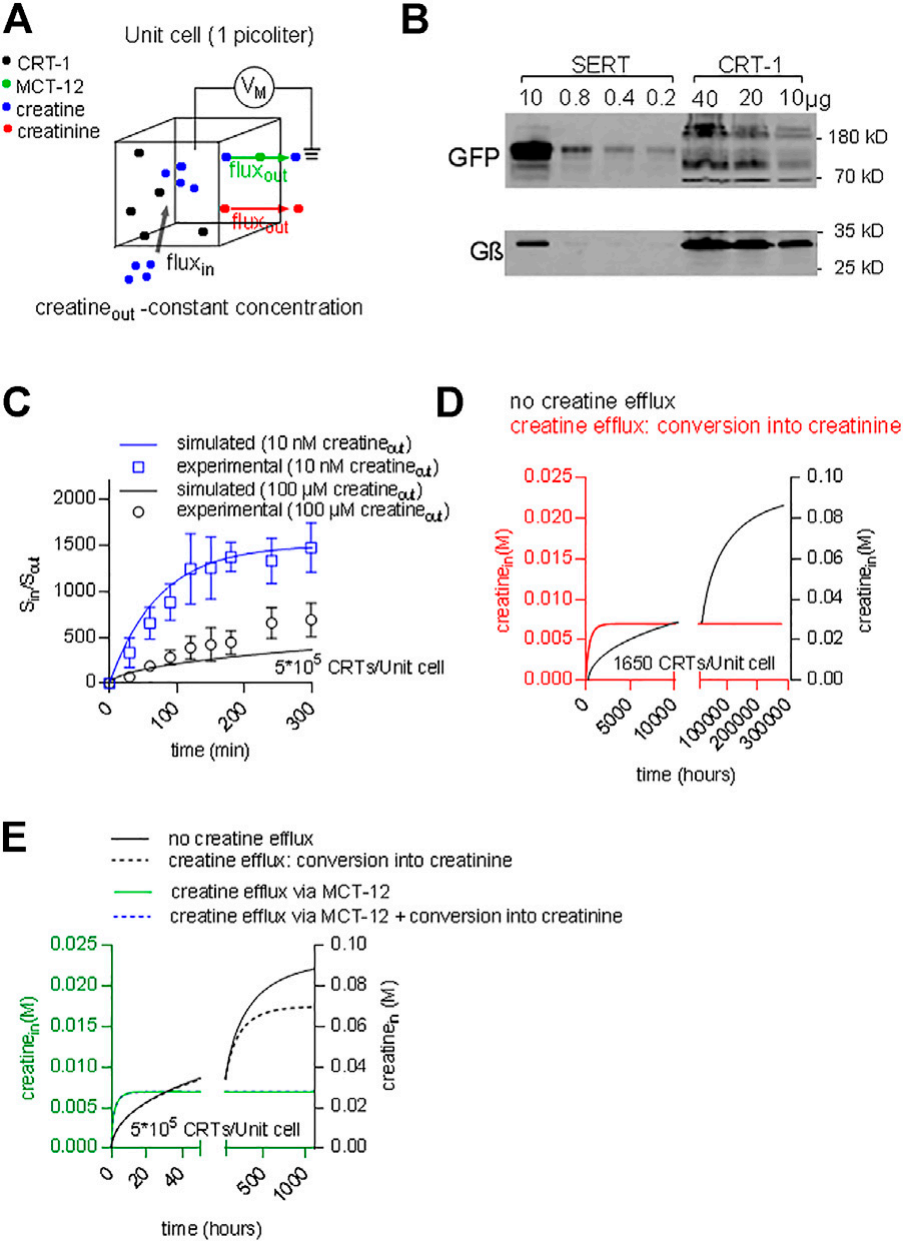
Predicting time-dependent accumulation of creatine. **(A)** A hypothetical unit cell is depicted with a volume of 1 picoliter. The model posits that creatine (blue dots) can enter the interior of the cell only via CRT-1 (black dots; black arrow) and that intracellular creatine can escape from its confinement via two routes: 1) conversion into creatinine (red dots) and subsequent diffusion through the cellular membrane (red arrow) and 2) outwardly directed transport of creatine via MCT-12 (green dots; green arrow). **(B)** Immunoblotting of CRT-1 transiently expressed in HEK293 cells: the membrane expression levels of YFP-tagged CRT-1 were compared to those in HEK293 cells, which had been stably transfected with a plasmid driving the expression of GFP-tagged SERT. The indicated amounts of membrane proteins were applied onto a denaturing gel, electrophoretically resolved and transferred to nitrocellulose. The transporter levels were detected by an antibody recognizing GFP and YFP; as a loading control, G protein β-subunits were detected with an antibody directed against an N-terminal sequence present in all G protein β-subunits. **(C)** The ratio of S_in_/S_out_ (i.e., concentrative power) as a function of time: the time-dependent accumulation of creatine was determined in HEK293 cells, which had been transiently transfected with a plasmid driving the expression of CRT-1. The data points in blue and black are from uptake experiments in which the extracellular [^3^H]creatine concentration was 10 nM and 100 μM, respectively. The solid lines are the corresponding predictions from a version of the model, where MCT-12 was omitted, because it is absent from HEK293 cells. **(D)** The accumulation of intracellular creatine over time was simulated for a unit cell containing 1650 CRT-1 molecules. In the simulations, creatine was assumed to be absent from the cell prior to time = 0. Exposure to a physiological extracellular creatine concentration (i.e., 30 µM) resulted in cellular uptake via CRT-1. The black line shows the exceedingly slow time course of the intracellular creatine concentration, if there is not any escape route for creatine. The red line corresponds to the time course defined by a model, which allows for conversion of creatine into creatinine. This creatine sink results in accelerated equilibration. If 1650 CRT-1 molecules/unit cell are present, the intracellular steady state concentration, which is present in muscle (i.e. 7 mM), is reached, but this still requires 3,000 h. **(E)** The accumulation of intracellular creatine over time was simulated for a unit cell, which contained 5*10^5^ CRT-1 molescules (solid and dashed black curve) and, in addition, MCT-12 (green line and dashed blue line). The solid and dashed black lines show the time course of creatine uptake without and with conversion to creatinine, respectively. The green line was generated by a model, where creatine uptake by CRT-1 occurred in the presence of MCT-12. The turnover number of MCT-12 for creatine is not known. Accordingly, the flux through MCT-12 was adjusted to yield a steady state intracellular creatine concentration of 7 mM. If conversion of creatine to creatinine was introduced as an additional sink for intracellular creatinine (dashed blue line), there wasn’t any appreciable effect on the time course of creatine accumulation.

The concentrative power of a secondary active transporter determines the concentration of substrate, which can be accumulated within a cell. The concentrative power provides another acid test to verify, if the model of the transport cycle provides an adequate description of the driving force for substrate influx. Inward transport is progressively slowed by accumulated substrate. Thus, the time-dependent approach to equilibrium depends on the initial concentration of substrate in the extracellular space. If our model of the transport cycle was realistic, it ought to allow for calculating both, the concentrative power and the time required to reach equilibrium. We assumed a cell with an intracellular volume of 1 picoliter surrounded by a membrane containing a given density of CRT-1 (black dots in Figure 10A). Cells vary considerably in size. This difference is however, immaterial to the computation, because the results obtained for this unit cell can be readily scaled up to the actual cell-volume of any given cell. We posited that creatine (blue dots) entered the cell only via CRT-1 (black dots; black arrow) and that there were two exit routes for intracellular creatine: 1) conversion into creatinine (red dots) and subsequent efflux (red arrow) and 2) efflux of creatine via the monocarboxylate transporter-12/MCT-12/SLC16A12 (green dots; green arrow) (Abplanalp et al., 2013; Takahashi et al., 2020). We ignore the conversion to intracellular conversion to phophocreatine, because it does not provide an exit route from the cell. We first examined the predictions in HEK293 cells. The average intracellular water space of a HEK293 cell is 1.25 pL (Sitte et al., 2001). HEK293 cells do not express MCT-12 (Takahashi et al., 2020); hence, the only exit route is the non-enzymatic conversion of creatine to creatinine. Approximately 1.7% of the creatine is converted into creatinine within 24 h (Wyss and Kaddurah-Daouk, 2000). This translates into a conversion rate of 2.3*10^–7^ s^−1^. The number of transporter molecules was estimated by immunoblotting with membranes harboring GFP-tagged SERT as the reference standard: the number of SERT molecules can be determined by radioligand binding; in the cell line stably expressing GFP-tagged SERT, the transporters accumulates to a level of 40 pmol/mg (Esendir et al., 2021); the primary antibody detects all variants of *Aequorea victoria* GFP (including YFP) with equal reactivity. Hence, GFP-SERT reactivity can be used to gauge CRT-1-YFP levels, because YFP and GFP are detected with equal efficiency. Based on immunoblotting (Figure 10B) we estimated that 10 µg membranes prepared from HEK293 transiently expressing CRT-1 contained immunoreactivity equivalent to that found in 0.8 µg of membranes harboring GFP-SERT. When corrected for cell number and transfection efficiency, this yielded a final estimate of about 5*10^5^ CRT-1/cell. We used these numbers (i.e. the creatine/creatinine sink and the transporter levels) to calculate the time course using the model outlined in Figure 7A with coop1 = 500 and coop 2 = 1 and to thereby estimate the concentrative power (i.e. the ratio of internally accumulated over extracellular creatine concentration). Under these assumptions, our calculations showed that the time required to reach thermodynamic equilibrium was substantially slower at high than at low substrate concentration (apparent rates 0.0013 and 0.0136 h^−1^, respectively) (Figure 10C). In contrast, the concentrative power was independent of the concentration. We verified these predictions by measuring the time-dependent accumulation of creatine in transfected HEK293 cells over the time period, which was accessible to an experimental study (i.e. over 300 min): the experimental data points, in particular for incubations using 10 nM external [^3^H]creatine (blue line and blue circles in Figure 10C) were reasonably close to the curves generated by the model (Figure 10C). Thus, the ternary complex model yields the concentrative power (about 1.5*10^3^), which is determined by the thermodynamic equilibrium, and the experimental observations are also consistent with the predictions.

In people, the extracellular creatine concentration is in the range of 15–120 µM (Arias et al., 2006). Accordingly, depending on the ionic gradients and the membrane potential, cells expressing CRT-1 ought to be able to concentrate creatine up to an intracellular concentration of about 0.06–0.5 M. In contrast to this prediction, the intracellular concentration of creatine in muscle cells was shown to reach only approximately 7 mM (Kushmerick et al., 1992). This discrepancy can be explained as follows: in a cell, the cytosolic concentration of a polar solute is determined by the rates of two fluxes: 1) the rate of cellular solute uptake via dedicated membrane transporters and 2) the rate by which the solute is removed from the intracellular pool. A steady state level of intracellular creatine is reached, if the rate of influx corresponds to the rate of removal, i.e. by conversion of creatine into creatinine. We surmised that in muscle cells these fluxes were balanced such that they did not allow for the cytosolic creatine concentration to exceed 7 mM. We verified this conjecture by resorting to the model of the unit cell described above (Figure 9A) to predict the cytosolic concentration of creatine within a muscle cell. For the simulation, we first assumed that 1) CRT-1 is the sole entry port for creatine into the unit cell and 2) that creatine is removed from the intracellular pool via non-enzymatic conversion into creatinine and its subsequent loss by diffusion through the cellular membrane (see above). In iterative cycles of calculations, we determined that 1,650 transporters/unit cell were required to maintain an intracellular concentration of 7 mM, if conversion of creatine into creatinine was the only exit route. Figure 10C illustrates the time-dependent accumulation of creatine, if this unit cell is exposed to 30 µM extracellular creatine (Figure 10C): in the absence of any conversion of creatine into creatinine, the intracellular creatine concentration was first requires the unrealistic time of 34 years to equilibrate (black curve in Figure 10D). Conversion of creatine (at a rate of 2.3*10^–7^ s^−1^) resulted in equilibration at 7 mM internal creatine (red curve in Figure 10D). However, the equilibrium was only reached after some 8 weeks (k = 0.0022 h^−1^). Thus, a unit cell can compensate for the constant loss of creatine and thus maintain an intracellular concentration of 7 mM creatine at steady state with 1650 CTR-1 molecules. However, the time course is unrealistic. It is not possible to remedy the slow equilibration by raising the expression level of CRT-1: In a unit cell, which harbors 5*10^5^ molecules, the time course is essentially the same and, importantly, creatine accumulates to a steady state concentration of about 70 mM, if conversion to creatinine is allowed to occur (dashed black line, in Figure 10E). Thus, reasonably rapid equilibration at 7 mM intracellular creatine cannot be achieved solely with two components (i.e. creatine influx via CTR-1 and the constant creatine/creatinine sink). In contrast, intracellular creatine can be readily clamped at 7 mM by introducing MCT-12 as creatine efflux transporter (Abplanalp et al., 2013; Takahashi et al., 2020): the rate for intracellular equilibration of creatine is accelerated 220-fold (0.48 h^−1^; green curve in Figure 10E), which allows a steady state to be reached within about 6 h. Under these conditions, the creatine sink resulting from its conversion to creatinine is immaterial for the intracellular steady state concentration and for the time course (dashed blue line in Figure 10E).

## Discussion

SLC6 transporters share a common mechanism of substrate translocation, but there are substantial variations in the partial reactions of the transport cycle: turnover numbers can, for instance, vary by more than an order of magnitude from less than 1 s^−1^ in the norepinephrine transporter NET/SLC6A2 (Bhat et al., 2021b) to 50 s^−1^ in the amino acid transporter SLC6A14 (Shi et al., 2021) Similarly, even the very closely related monoamine transporters - i.e. the transporters for dopamine, serotonin and norepinephrine - differ in their ability to harvest the potassium gradient or the membrane potential (Bhat et al., 2021b). These differences presumably reflect adaptations to physiological requirements (Schicker et al., 2022). Here, we relied on the high temporal resolution provided by electrophysiological recordings to analyze the transport cycle of the creatine transporter. Our observations show that 1) CRT-1 can harness both, the gradient of co-substrate ions - i.e. of sodium and chloride - and the membrane potential, but 2) that potassium is immaterial to the transport cycle. 3) All known competitive inhibitors, i.e. β-GPA, ATPCA and cyclocreatine, are full alternative substrates. This conclusion is based on two findings: they elicited transport-associated currents, which were comparable in magnitude to those triggered by the cognate substrate creatine and which deactivated with virtually identical kinetics. Thus, they were translocated with similar turnover rates. 4) CRT-1 binds the substrate and the co-substrate ions in a highly cooperative manner. This cooperativity allows for continuous cycling of CRT-1 in the forward transport mode in spite of a rise in intracellular creatine concentration. Thus, CRT-1 is engineered to exploit its concentrative power, because it escapes the brake imposed by intracellular substrate accumulation and does not switch into the reverse or exchange mode.

CRT-1 and GlyT1 share the same transport stoichiometry and they both bind (co)-substrates in a cooperative manner. Here we show that the mechanisms of cooperativity of CRT-1 differs from that previously described for GlyT1 (Erdem et al., 2019). In GlyT1, destabilization of the quaternary complex occurs upon dissociation of only one (co)-substrate, i.e. of sodium, chloride or glycine. In contrast, in CRT-1, two (co)-substrates must be released to achieve disassembly of the quaternary complex. The calculations based on the kinetic model show that this subtle mechanistic difference is the most plausible explanation to account for the ability of CRT-1 to operate at full velocity in the presence of millimolar intracellular creatine concentrations. The kinetic model, which we proposed, has some simplifications: most notably, it treats binding of the two sodium ions as a single step reaction. Many SLC6 transporters form oligomers (Schmid et al., 2001; Chiba et al., 2014); SERT oligomers are thought to exchange rapidly in the ER but to be stable in the plasma membrane (Anderluh et al., 2014a,b). The oligomeric state of CRT-1 is not known, but our immunoblots indicate that these may exist. For the sake of parsimony, we did not consider possible differences between transport kinetics of monomeric and oligomeric CRT-1. Nevertheless, the model allowed for recapitulating essentially all experimental observations. Importantly, the model also offered insights into the requirement for creatine efflux. The non-enzymatic conversion of creatine to creatinine acts as a constant sink (Borsook and Dubnoff, 1947). Our calculations show that a very modest expression level of CRT-1 (i.e., 1,650 molecules of CRT-1/unit cell) suffices to compensate for this constant loss. However, with this complement of transporter molecules, it is impossible to reach an equilibrium within a biologically relevant time frame: this is evident, if proliferating cells are considered: transit through the G1 phase of the cell cycle halves the intracellular creatine level, which must be replenished before the next cell cycle is initiated. It is possible to remedy the speed of creatine accumulation by raising the expression levels of CRT-1 substantially. This, however, overcompensates the creatine/creatinine sink and clamps the steady state levels of intracellular creatine in the vicinity of the concentration dictated by the thermodynamic equilibrium. Thus, a major insight arising from our calculations is the finding that an additional creatine efflux mechanism - other than the creatinine sink - is inevitable: it is required to explain how e.g. skeletal muscle and neurons accumulate intracellular creatine to steady-state level of about 7 mM (Kushmerick et al., 1992) and about 5 mM (Rackayova et al., 2017), respectively. In fact, MCT-12 is expressed in both, skeletal muscle and in the brain (Abplanalp et al., 2013) and MCT-12 can mediate efflux of creatine (Takahashi et al., 2020). MCT-12 is present in the sinusoidal membrane compartment of hepatocytes (Jomura et al., 2021) and in the basolateral membrane compartment renal tubular epithelium (Dhayat et al., 2016). The physiological role of MCT-12 in these compartments can readily be appreciated: most mammalian cell types lack the capacity to synthesize creatine and rely on the supply by hepatic synthesis, which utilizes guanidino-acetate provided by the kidney (Wyss and Kaddurah-Daouk, 2000). MCT-12 in the liver and kidney account for the bulk of released creatine (Jomura et al., 2021) and guanidino-acetate (Dhayat et al., 2016), respectively. In contrast, the abundant expression of MCT-12 in brain, heart and skeletal muscle (Abplanalp et al., 2013) is difficult to rationalize, unless the predictions of our calculations are taken into account. We conclude from our analysis that the presence of MCT-12 is required to maintain creatine at a steady state level below the intracellular concentration specified by the thermodynamic equilibrium of CRT-1.

The monocarboxylate transporter-9 (MCT-9/SLC16A9) has been proposed as an additional creatine efflux transporter (Futagi et al., 2020; Jomura et al., 2021). MCT-9 is a low-affinity transporter, which operates as a H^+^/creatine exchanger with biphasic transport kinetics: K_M_ values of about 24 and 240 mM have been reported for the two components (Futagi et al., 2020). Because of this poor affinity and because the proton gradient points in the wrong direction, MCT-9 is unlikely to contribute substantially to cellular creatine efflux. In contrast, the K_M_ of MCT-12 is in the range of 0.3 mM (Futagi et al., 2020) to 0.57 mM (Abplanalp et al., 2013). Hence, with intracellular creatine levels in the range of 5–7 mM, MCT-12 operates under saturating conditions. Accordingly, our model posits that the intracellular concentration of creatine, which is maintained at equilibrium in a given cell, is determined by the concentrative power of CTR-1 and the expression level of MCT-12. We concede that this is a simplified model, because the activity of either transporter may be subject to regulation and there may be transporters other than MCT-12, which support creatine efflux. In fact, MCT-12 apparently accounts only for 75% of creatine efflux from liver (Jomura et al., 2021).

Inactivating mutations in CRT-1 lead to an X-linked syndrome of intellectual disability, impaired language acquisition, autism and epileptic seizures. There are large variations in the manifestations of human CRT-1 deficiency (van de Kamp et al., 2014; Farr et al., 2020). Consistently, however, symptoms predominate which arise from impaired neuronal function. In contrast, skeletal muscle or cardiac function is not or only mildly impaired. In fact, creatine levels in skeletal muscle are normal in the limited number of patients with CRT-1 deficiency, in whom these have been assessed (DeGrauw et al., 2003; Pyne-Geithman et al., 2004). In contrast, brain levels of creatine are substantially reduced in CRT-1 deficiency (Rackayova et al., 2017). Human skeletal muscle may compensate for CRT-1 deficiency by increased creatine synthesis (Russell et al., 2014; van de Kamp et al., 2014; Stockebrand et al., 2018). In contrast, in the brain, the enzymes involved in creatine synthesis (i.e. L-arginine:glycine amidinotransferase/AGAT and guanidinoacetate methyltransferase/GAMT) are expressed in different cellular compartments (Braissant and Henry, 2008). Thus, shuttling - i.e. release and uptake - of precursors and of creatine via their cognate transporters is important to maintain intracellular creatine levels in neurons (Braissant and Henry, 2008). The levels of creatine in the cerebrospinal fluid is within the normal range (15–90 µM) in patients with CRT-1 deficiency (Cecil et al., 2001; DeGrauw et al., 2002; van der Kamp et al., 2103). In the absence of any functional CRT-1, the blood-brain barrier is impermeable to creatine. Hence, creatine in the cerebrospinal fluid must originate from brain cells, which release creatine. In hippocampal neurons, which were preloaded with radiolabelled creatine, sustained, carrier-mediated release of creatine can be unmasked by omitting extracellular sodium (and thus inhibiting reuptake by CTR-1) (Dodd et al., 2010). This turnover appears to be substantial: more than 50% of preloaded creatine is released within 1 h (Dodd et al., 2010). This release is presumably due to the action of MCT-12. A vexing problem in creatine transporter deficiency is to understand the phenotypic consequence of inactivating mutations in CRT-1. The manifestation can vary even within a family: in three brothers, who harbored the folding-deficient G424D mutation (El-Kasaby et al., 2019), the clinical course differed substantially with mild disease in two siblings and full-fledged manifestation of the disorder in the third (Puusepp et al., 2010). This variation in expressivity can only be accounted for by the action of disease-modifying genes. Accordingly, based on our insights, we argue that the interplay between CRT-1 and MCT-12 is worth examining to understand the clinical manifestations of creatine transporter deficiency.

## Data Availability

The original contributions presented in the study are included in the article/supplementary material, further inquiries can be directed to the corresponding authors.
